# A randomized placebo−controlled clinical trial of oral green tea epigallocatechin 3−gallate on erythropoiesis and oxidative stress in transfusion−dependent β−thalassemia patients

**DOI:** 10.3389/fmolb.2023.1248742

**Published:** 2024-01-24

**Authors:** Kornvipa Settakorn, Sasinee Hantrakool, Touchwin Petiwathayakorn, Nuntouchaporn Hutachok, Adisak Tantiworawit, Pimlak Charoenkwan, Nopphadol Chalortham, Anchan Chompupoung, Narisara Paradee, Pimpisid Koonyosying, Somdet Srichairatanakool

**Affiliations:** ^1^ Department of Biochemistry, Faculty of Medicine, Chiang Mai University, Chiang Mai, Thailand; ^2^ Division of Hematology, Department of Internal Medicine, Faculty of Medicine, Chiang Mai University, Chiang Mai, Thailand; ^3^ Thalassemia and Hematology Center, Faculty of Medicine, Chiang Mai University, Chiang Mai, Thailand; ^4^ Division of Hematology and Oncology, Department of Pediatrics, Faculty of Medicine, Chiang Mai University, Chiang Mai, Thailand; ^5^ Department of Pharmaceutical Sciences, Faculty of Pharmacy, Chiang Mai University, Chiang Mai, Thailand; ^6^ Royal Project Foundation, Chiang Mai, Thailand

**Keywords:** green tea, epigallocatechin-3-gallate, thalassemia, iron overload, erythropoiesis, erythroferrone

## Abstract

β−Thalassemia patients suffer from ineffective erythropoiesis and increased red blood cell (RBC) hemolysis. Blood transfusion, erythropoietic enhancement, and antioxidant supplementation can ameliorate chronic anemia. Green tea extract (GTE) is comprised of catechin derivatives, of which epigallocatechin−3−gallate (EGCG) is the most abundant, presenting free−radical scavenging, iron−chelating, and erythropoiesis−protective effects. The present study aimed to evaluate the effects of GTE tablets on the primary outcome of erythropoiesis and oxidative stress parameters in transfusion−dependent β−thalassemia (TDT) patients. Twenty−seven TDT patients were randomly divided into placebo and GTE tablet (50 and 100 mg EGCG equivalent) groups and assigned to consume the product once daily for 60 days. Blood was collected for analysis of hematological, biochemical, and oxidative stress parameters. Accordingly, consumption of GTE tablets improved blood hemoglobin levels when compared with the placebo; however, there were more responders to the GTE tablets. Interestingly, amounts of nonheme iron in RBC membranes tended to decrease in both GTE tablet groups when compared with the placebo. Importantly, consumption of GTE tablets lowered plasma levels of erythroferrone (*p <* 0.05) and reduced bilirubin non−significantly and dose−independently. Thus, GTE tablets could improve RBC hemolysis and modulate erythropoiesis regulators in transfusion−dependent thalassemia patients.

## 1 Introduction

Ineffective erythropoiesis (IE) in β−thalassemia patients is genetically caused by the absence or diminished production of β−globin chains, leading to an excess and precipitation of α−globin chains, as well as oxidative stress in erythroid precursors (Cao and Galanello, 2010; Galanello and Origa, 2010). In addition, reduced erythroid cell differentiation and survival, together with increased red blood cell (RBC) hemolysis, will worsen the anemia (Rivella, 2019). A high increase of duodenal iron absorption is predominantly found in non-transfusion−dependent β−thalassemia (NTDT) patients, while repeated blood transfusions are required for transfusion−dependent β−thalassemia (TDT) patients to compensate for the, IE and maintain normal blood hemoglobin (Hb) levels (Rivella, 2012; Taher et al., 2018). Consequently, secondary iron overload induces oxidative tissue damage and escalates the mortality in these patients (Taher and Saliba, 2017). Nowadays, iron chelators, such as desferrioxamine (DFO), deferiprone (DFP), and deferasirox (DFX), are being used for the treatment of β−thalassemia patients with iron overload, even when showing adverse effects (Viprakasit and Origa, 2014; Cappellini and Motta, 2017). Recently, novel therapeutics for β−thalassemia patients involving Janus kinase (Jak) inhibitors, hepcidin agonists, such as minihepcidin and transmembrane serine protease 6 (TMPRSS6) antagonist, and ferroportin inhibitors, such as VIT−2763 and apo−transferrin, have been reported to improve iron dysregulation (Rivella, 2012; Gelderman et al., 2015; Makis et al., 2021). Interestingly, allogeneic hematopoietic stem cell transplantation involving gene therapy with γ− or β−globin insertion, transformed growth factor−β ligand traps, such as luspatercept, pyruvate kinase activators, such as mitapivat, and fetal hemoglobin (HbF) inducers, such as phosphodiesterase 9 inhibitor tovinontrine (IMR−687) and resveratrol, have been reported to restore normal erythropoiesis (Theodorou et al., 2016; Makis et al., 2021; Matte et al., 2021).

Erythropoietin (EPO) is synthesized by the kidneys to activate marrow erythrocytic progenitors for RBC synthesis and to respond to hypoxia (Amer et al., 2010). Orchestrally, erythroferrone (ERFE) is an erythroid regulator secreted by erythroblasts in response to EPO activation and the repression of hepcidin synthesis by the liver to mediate iron overload (Kautz and Nemeth, 2014). In NTDT patients, high amounts of ERFE from the expanded pool of immature erythroid cells can suppress hepcidin expression and production, and consequently exacerbate their, IE and anemia (Theodorou et al., 2016; Pagani et al., 2019). In NTDT patients, where extramedullary erythropoiesis was commonly found, levels of serum ferritin, growth differentiation factor 15 (GDF15), and EPO were significantly increased, while serum levels of ERFE, hepcidin, and soluble transferrin receptors (sTfR) were not significantly changed (Huang et al., 2019). Under, IE of β-thalassemia intermedia (TI) mice, ERFE mRNA levels were significantly increased in both the marrow and spleen, while large amounts of ERFE protein were elevated in the plasma, and liver hepcidin expression and production levels were inhibited. Consequently, this resulted in increases of duodenal iron absorption, plasma iron levels, and liver iron contents (LIC) (Kautz et al., 2015). More importantly, higher serum hepcidin levels were found in β-thalassemia major (TM) and TI patients than for the thalassemia trait (TT). Accordingly, lower hepcidin/ferritin ratios were observed among the TM patients than in the TI and healthy groups, and higher serum GDF15 levels were recorded in TM and TI patients than in the TT and healthy groups. Moreover, higher ERFE concentrations and EPO activities were observed in the TI groups than in the TM, TT, and healthy groups (Ozturk et al., 2021).

Green tea (*Camellia sinensis*) extract (GTE) contains catechins, gallocatechin 3−gallate, (-)−epicatechin (EC), (-)−epigallocatechin (EGC), (-)−epicatechin−3−gallate (ECG), and (-)−epigallocatechin−3−gallate (EGCG) (Chacko et al., 2010; Khan and Mukhtar, 2013), which can be analyzed effectively using high−performance liquid chromatography equipped with a diode array detector (HPLC−DAD) in conjunction with electrospray ionization−mass spectrometry (HPLC/ESI−MS) and ultraHPLC/ESI−MS time of flight (UHPLC/ESI−TOF/MS) (Clarke et al., 2014; Dai et al., 2017; Koonyosying et al., 2018). Interestingly, EGCG, as the most abundant compound, possesses antioxidant, anticancer, radical−scavenging, anti−inflammatory, and cardiovascular−protective properties (Chacko et al., 2010; Khan and Mukhtar, 2018). In addition, the compound can remove excessively−accumulated iron in tissues, redox−active iron in the plasma and RBC membrane (Thephinlap et al., 2007), and relieve hemolysis of thalassemia RBC (Saewong et al., 2010). Importantly, treatments of GTE diminished the levels of plasma EPO and ERFE, while consistently suppressing kidney *Epo* and spleen *Erfe* mRNA expressions to a significant degree in iron-loaded BKO mice when compared with untreated mice, and the treatments also decreased plasma ferritin levels, along with iron content levels in the liver (*p* < 0.05), spleen (*p* < 0.05), and kidney tissues of iron loaded BKO mice (Settakorn et al., 2022). Furthermore, lipid-peroxidation products in the tissue and plasma were also decreased when compared with the untreated mice (Settakorn et al., 2022). Thus, manipulations of iron metabolism, erythropoietic activity, or erythroid cell differentiation, and survival could improve iron overload and anemia in β−thalassemia patients. Hypothetically, EGCG−rich GTE should enhance erythropoiesis, ameliorate oxidative RBC, and prolong RBC survival in β−thalassemia patients. The present study aimed to investigate whether consumption of GTE could effectively influence levels of erythropoiesis and oxidative stress parameters in TDT patients.

## 2 Materials and methods

### 2.1 Chemicals and reagents

Standard EGCG was purchased from Aktin Chemical Inc., Chengdu, PR. China. Standard quercetin and bovine serum albumin (BSA) were obtained from Sigma−Aldrich Chemical Company, Saint Louis, MO, United States. Accordingly, 2,2′−azo−bis(2−amidinopropane) dihydrochloride (AAPH), phosphoric acid (H_3_PO_4_), sodium dithionite, and trichloroacetic acid (TCA) were purchased from Merck−Millipore Group, Merck KGaA, Darmstadt, Germany. Ammonium chloride, 2,2−diphenyl−1−picrylhydrazyl (DPPH), 2,2'−azinobis−(3−ethylbenzothiazoline−6−sulphonic acid) diammonium salt (ABTS), L−ascorbic acid (AA), butylated hydroxytoluene (BHT), ferrous ammonium sulfate (FAS), 6−hydroxy−2,5,7,8−tetramethylchroman−2−carboxylic acid (Trolox), 4−morpholinepropanesulfonic acid (MOPS), potassium persulfate (K_2_S_2_O_8_), sodium dodecylsulfate (SDS), Bradford reagent, 3−(2−pyridyl)−5,6−diphenyl−1,2,4−triazine−*p,p′*−disulfonic acid monosodium salt hydrate (ferrozine), Triton−X100, phosphate buffered saline (PBS) pH 7.4, and thiobarbituric acid (TBA) were purchased from the Sigma−Aldrich Chemical Company (Saint Louis, MO, United States). All organic solvents (HPLC or highest pure grade) were purchased from Thermo Fisher Scientific Inc., Waltham, MA, United States. Milli−Q ultrapure deionized (DI) water was purchased from Merck−Millipore Group, Merck KGaA, Darmstadt, Germany. White−colored microcrystalline cellulose pH101 (MCC) powder (Product number 435236, pH 4.5–7.5, particle size distribution +200 Mesh ≥40%) and polyvinylpyrrolidone K90 (PVP K90) (Product number 81440) were also purchased from the Sigma−Aldrich Chemical Company (Saint Louis, MO, United States).

### 2.2 Preparation of GTE powder

GTE was prepared using the method established by Koonyosying and others (Koonyosying et al., 2018). Briefly, fresh tea shoots were harvested from tea fields of the Royal Project Foundation at Mon−ngao, Amphur Maetaeng, Chiang Mai Province and immediately dried at 100°C for 3 min in an electric microwave oven (Electrolux, Stockholm, Sweden, 20−L capacity, 4,000−watts, 220 V electric power) to inactivate inherent polyphenol oxidase. Dry tea leaves were converted to a powder with an electric blender (SharpThai Company, Limited, Thailand), and the tea powder (1 kg) was extracted in 10 L of hot DI (80°C) for 10 min and ultrafiltrated through a filter membrane (cellulose acetate type, 50 mm diameter, 0.45 µm pore size, GE Healthcare Life Sciences, Whatman, Maidstone, Kent, United Kingdom) under a vacuum. Finally, GTE filtrate was mixed with maltodextrin (5%, *w*/*v*) and dried using a spray dryer (T.S.K. Engineering Company, Limited, Chonburi, Thailand). Spray−dried GTE granules were then packed in an aluminum foil bag (10 kg capacity) and kept at 4°C in a refrigerator until being used.

### 2.3 Preparation of GTE tablets

Spray−dried GTE tablets were prepared using the wet granulation method established by Kristensen and Hansen with slight modifications (Kristensen and Hansen, 2006). At first, GTE granules were gradually added to a binding solution comprised of MCC (2:1, *w/w*) and PVP K90 (5%, *w*/*w*) until the homogenous PVP K90 solution reached a final concentration of 1% (*w*/*w*). Then, the mixture was passed through a nylon net filter (size 14 mesh) and dried with a fluid−bed dryer for 5 min until GTE granules were formed. Afterwards, dry GTE granules were compressed into oval−shaped tablets of 750 mg weight and 50 mg EGCG equivalent, while a placebo tablet was prepared similarly containing all ingredients except for GTE. Accordingly, both products had the same shape, size, and color as the GTE tablets. Placebo and GTE tablets were packaged in white polypropylene bottles (30 tablets each) with a sealed cap and stored at 4°C in a refrigerator until being used.

### 2.4 HPLC−DAD quantification of catechins

An EGCG sample derived from GTE and spray−dried GTE tablets was quantified using isocratic reverse−phase HPLC−DAD (Srichairatanakool et al., 2006; Thephinlap et al., 2007). Briefly, the exact GTE (10 mg) and spray-dried GTE tablet (50 mg) were reconstituted in 1 mL of DI water and passed through a syringed membrane filter (polytetrafluoroethylene type, 0.45−μm pore size, 13−mm diameter, Monotaro Company, Limited, Tokyo, Japan). Then, 20 µL of the GTE solution (10 mg/mL) and the GTE tablet solution (50 mg.mL) was injected into the HPLC−DAD system (Model 1290 Infinity II, Agilent Technologies, Inc., Santa Clara, CA, United States) and fractionated on the column (ODS type, 150 mm × 4.6 mm, 5 µm particle size, Agilent Technologies, Inc., Santa Clara, CA, United States) capped with a guard column (10 mm × 4.7 mm, 5 µm particle size, Agilent Technologies, Inc., Santa Clara, CA, United States). Individual catechins were eluted isocratically with a mobile−phase solvent containing 0.05% H_2_SO_4_: acetonitrile: ethyl acetate (86: 12: 2, *v/v/v*) at a flow rate of 1.0 mL/min, and optical density (OD) was detected at 280 nm with DAD. The standard curve for EGCG was obtained by using the peak areas of five different concentrations; 0.625, 1.25, 2.5, 5, and 1 mg/mL, that were obtained by dissolving the EGCG reference standard in DI water.

### 2.5 UHPLC/ESI−QTOF/MS analysis of catechins

Phenolic compositions of GTE tablets and their plasma metabolites were analyzed using the comprehensive UHPLC/ESI−QTOF/MS method previously described by Hodgson and others (Hodgson et al., 2014) with slight modifications at the Central Laboratory, Faculty of Agriculture, Chiang Mai University, Chiang Mai, Thailand (Prommaban et al., 2020). The UHPLC/ESI−QTOF/MS system was composed of an HPLC machine (Agilent 1260 Infinity II LC, Agilent Technologies, Inc., Santa Clara, CA, United States) equipped with an ESI−QTOF/MS. In the MS system, nitrogen gas nebulization was set at 45 pounds per inch^2^ with a flow rate of 5.0 L/min at 300°C, the sheath gas was set at 11.0 L/min at 250°C, and the capillary and nozzle voltage values were set at 3.5 kV and 500 V, respectively. A complete mass scan was conducted as a mass−to−charge ratio (m/z) ranging from 200 to 3200. All the operations, acquisition, and analysis of the data were monitored using Agilent UHPLC−QTOF−MS MassHunter Acquisition Software Version B.04.00 “Find by Be” algorithm to generate a list of precise mass matches−compounds. Peak identification was performed in positive modes using the library database, and the identification scores were further selected for characterization and m/z verification.

In the sample preparation, GTE tablets and plasma were extracted in a mixture of DI water (300 µL), 2 M HCl (10 µL), and ethyl acetate (1.0 mL) and then spun in a refrigerated benchtop microcentrifuge (MIKRO 185, Andreas Hettich GmbH & Co. KG, Föhrenstrasse, Tuttlingen, Germany) at 2,700 g for 10 min at room temperature. The ethyl acetate layers were collected, pooled, mixed with 0.4% ascorbic acid (5 µL), and dried under N_2_ gas flow at 6–10 L/min. Then, the powder was reconstituted in a mixture of absolute methanol (100 µL) and Milli Q water (100 µL), ultrasonicated on an ice bath for 10 min, and centrifuged at 17,000 g, room temperature for 10 min. Afterward, the supernatant was passed through an acrodisc syringe filter (cellulose ester type, 0.45 µm pose size, Monotaro Company, Limited, Tokyo, Japan). In the analysis, the filtrate (5 μL) was injected into the HPLC system using an autosampler and fractionated on a column (InfinityLab Poroshell 120 EC octadecyl silane type, 2.1 mm × 100 mm, 2.7 µm particle size, Agilent Technologies Company, Santa Clara, CA, United States) that had been thermally regulated at 40°C and eluted in a linear gradient mode using mobile phase A (0.1% formic acid in DI) and mobile phase B (0.1% formic acid in acetonitrile) at a flow rate of 0.35 mL/min for 60 min. The timing program employed for gradient elution was as follows: 0→15 min: %A/B (100/0→90/10), 15→30 min: %A/B (90/10→40/60): 30→45 min: A (40/60→10/90); and 45→60 min: A (10/90→0/100). Peak identification was carried out in positive mode using the library database, and the identification scores were sorted out for the purposes of characterization and m/z verification.

### 2.6 Determination of antioxidant activity

#### 2.6.1 DPPH assay

GTE tablets (750 mg) were crashed in ceramic cups, dissolved in PBS pH 7.0 solution to achieve EGCG concentrations of 25 and 50 mg/mL, and centrifuged at 2,700 g at room temperature for 10 min to remove sediment particulate. Trolox, also known as 6-hydroxy-2,5,7,8-tetramethylchroman-2-carboxylic acid, is a water-soluble analog of α-tocopherol, which is a powerful benchmark antioxidant that is employed in biochemical applications to measure antioxidant capacity *in vitro* and alleviate oxidative stress. Stock Trolox solution (2.5 mM) was freshly prepared by dissolving 3.13 mg of Trolox in 5 mL of absolute ethanol and was sequentially two−fold diluted with PBS before being used. In the assay, 1.0 mL of the GTE, Trolox solution (0–2.5 mM), and DI water were incubated with 1.0 mL of 0.4 mM DPPH solution at room temperature for 10 min, while OD was measured at 517 nm against the reagent blank using spectrophotometry (Paradee et al., 2019).

#### 2.6.2 ABTS assay

Firstly, 7.4 mM ABTS solution (1.5 mL) was incubated with 2.8 mM potassium persulfate solution (1.5 mL) at room temperature in a dark chamber for 12–16 h to form blue−colored ABTS cationic radical (ABTS^+•^), and the OD of the resulting ABTS^+•^ solution was adjusted at 734 nm with DI water to reach a value of 0.70 ± 0.02. In the assay, 10 µL of the GTE sample, Trolox solution (0–2.5 mM), and DI water were added to the working ABTS^•+^ solution (1.0 mL), incubated at room temperature for exactly 6 min, and the resulting OD value was measured at 734 nm against the reagent blank using spectrophotometry (Paradee et al., 2019).

### 2.7 Ethics statement

The study protocol was conducted in accordance with the Declaration of Helsinki and approved by the Research Ethical Committee for Human Study of Faculty of Medicine, Chiang Mai University, Chiang Mai, Thailand (Study code: MED−2561−05846). The study was also reviewed and approved by Thai Clinical Trials Registry (TCTR) committees, for which the TCTR identification number is TCTR20211118002. All patients gave their informed consent before their inclusion in the study. This study was conducted from 01/07/2020 to 31/05/2021 in accordance with the reporting guidelines of the Consolidated Standards of Reporting Trials (CONSORT) 2010 (Schulz et al., 2010).

### 2.8 Patients and study design

Transfusion−dependent thalassemia patients who regularly receive their physical and blood examinations at the Adult Thalassemia Clinic, Maharaj Nakorn Chiang Mai Hospital, Faculty of Medicine, Chiang Mai University, Chiang Mai, Thailand were enrolled in this study. Regarding the inclusion criteria, all subjects were Thai adult TDT patients aged 20–65 years, who could communicate in the Thai language, had visited the clinic for regular treatments, and who had not received any extra−treatments except for blood transfusions (Tx) and iron chelation therapy of DFO, DFX, GPO−L1 (a generic DFP), or a combination of any of them for at least 3 months before and during the course of the study. The exclusion criteria encompassed the following conditions: the presence of other significant hematological disorders, a documented history of substantial liver or kidney issues, ongoing infections or fever at the time of recruitment, being pregnant or breastfeeding, an inability to provide informed consent, and those who engaged in smoking or alcohol consumption during the course of the study period. Using stratified sampling, thirty−one subjects were recruited but three of them dropped out from the study. Accordingly, twenty−seven thalassemia patients were enrolled in this study and were randomly divided into three groups: group 1 receiving the placebo (*n* = 8), group 2 receiving the GTE tablet (50 mg EGCG equivalent) (*n* = 9), and group 3 receiving double the dosage of the GTE tablets (100 mg EGCG equivalent) (*n* = 10). The results of genotype and physical examinations, involving age, height, body weight (BW), body mass index (BMI), and liver and spleen palpability, were also recorded before initiating the study. All the subjects consumed the product once daily for 60 days and were asked to avoid consuming foods that were rich in polyphenolic compounds. Notably, blood samples were collected on days 0, 30, and 60 after stopping their iron chelation treatment for 72 h and before receiving their next blood transfusion. A flow−chart of patient preparation and intervention are shown in Supplementary Figure S1.

### 2.9 Blood collection

Venous blood samples were collected from patients who had fasted for 8–12 h, wherein one part (3 mL) was transferred to an EDTA−coated vacutainer tube for analysis of hematological parameters, and the other part (7 mL) was transferred to a heparin vacutainer tube. Heparinized blood was subjected to centrifugation at 1,500 g at 4°C for 20 min, while plasma was separated from blood cell sediment and kept in 1 mL aliquot cryotubes at −20°C until complete analysis of the biochemical parameters and erythropoiesis biomarkers was conducted.

### 2.10 Determination of RBC indices

EDTA blood was used for analysis of RBC indices, including RBC numbers, Hb, hematocrit (Hct), mean corpuscular volume (MCV), mean corpuscular hemoglobin (MCH), mean corpuscular hemoglobin concentrations (MCHC), RBC distribution width (RDW), and reticulocyte count at a Central Laboratory, Maharaj Nakorn Chiang Mai Hospital, Faculty of Medicine, Chiang Mai University, Chiang Mai, Thailand using an Automate Cell Counter (Model DxH900, Beckman Coulter, Brea, CA, United States) according to the manufacturer’s instructions.

### 2.11 Assay of Anti−Hemolysis activity

Packed RBCs from Section 2.9 were washed twice with PBS at a pH of 7.4 with centrifugation at 3,140 g for 5 min, while 10% RBC suspension was prepared in PBS at a pH of 7.4. The cell suspension was divided into three tubes (150 µL each), which were treated with 150 µL of PBS at a pH of 7.4 (negative), 1% Triton−X100 (positive), or 100 mM AAPH (test). In principle, AAPH is a soluble azo compound which will decomposes to generate 1 mol of nitrogen and 2 mol of carbon radicals, and subsequently cause RBC hemolysis (Banerjee et al., 2008; He et al., 2013). The specimens were then incubated at 37°C with gentle shaking at 450 g for 2 h. Blood samples obtained from healthy volunteers were analyzed similarly. After incubation, the hemolysates were centrifuged at 1,350 g for 10 min and the OD value was measured at 540 nm (Ngarmpattarangkoon et al., 2016). Accordingly, the percentage of anti−hemolysis activity was derived from the following equation of $\left\{\left({\text{OD}}_{\text{Negative}}\;-\;{\text{OD}}_{\text{Sample}}\right)/\left({\text{OD}}_{\text{Negative}}\;-\;{\text{OD}}_{\text{Positive}}\right)\right\}x100$ .

### 2.12 Measurements of EPO, ERFE, and hepcidin concentrations

Plasma human EPO, ERFE, and hepcidin concentrations were measured using sandwich enzyme−linked immunosorbent assay (ELISA) kits (Catalogue numbers: abx655612, abx258690, and abx251355, respectively) (Settakorn et al., 2022) according to the manufacturer’s instructions (Abbexa Company, Bar Hills, Cambridge, United Kingdom).

### 2.13 Measurement of nonheme iron content in RBC membrane

Heparinized blood was centrifuged at 3,140g, 4°C for 10 min and the plasma was removed. Packed RBCs were washed twice with normal saline solution (NSS) by centrifugation at 3,140 g for 5 min and 20% RBC suspension was prepared in NSS. Then, 500 µL of cell suspension was mixed and incubated with 1 mL of lysis buffer (0.1% Triton X−100 in NSS) at room temperature for 30 min. After centrifugation at 10,800 g for 5 min, the supernatant was discarded, while ghost RBC membrane pellets were washed twice with the lysis buffer and resuspended in 1.0 mL of 10 mM MOPS pH 7.0 buffer. For the colorimetric assay, 100 µL of the membrane suspension was incubated with 250 µL of 0.6% SDS in 0.2 M sodium acetate buffer at a pH of 4.5 for 10 min, with 125 µL of ascorbic acid reagent (3.0 g ascorbic acid and 0.1 g sodium metabisulfite in 50 mL 0.2 M sodium acetate pH 4.5 buffer) for 5 min, and with 25 µL of chromogenic ferrozine reagent (200 mg ferrozine and 1.25 g thiourea in 50 mL DI water) for 2 min consecutively at room temperature. Finally, the OD value of the resulting magenta colored Fe^2+^−ferrozine complex was measured at 570 nm (Srichairatanakool et al., 2013). Nonheme iron concentration was determined from a standard graph made from standard FAS solution (0–200 µM).

### 2.14 Measurement of TBARS content in RBC membrane and plasma

In the assay, 80 µL of the RBC membrane suspension obtained from Section 2.13, or the plasma, was mixed with 10 µL of 0.2% (*v*/*v*) BHT, 240 µL of 0.44 M H_3_PO_4_, and 160 µL of 0.6% TBA. It was then incubated at 90°C for 30 min and cooled down in an ice bath. Afterwards, the suspension was centrifuged at 3,000 g for 10 min. Finally, the supernatant was extracted to measure OD at 540 nm against the reagent blank using spectrophotometry. TMP, also referred to as 1,1,3,3–tetramethoxypropane, serves as a precursor for malondialdehyde and is a naturally occurring byproduct of lipid peroxidation. It was subsequently used as a reference standard for lipid−peroxidation products at a concentration ranging from 0 to 100 µM (Koonyosying et al., 2018).

### 2.15 Measurement of protein content in RBC membrane

RBC membrane suspension was dissolved in NSS (1:1, *v/v*) and mixed with Bradford reagent (100:1, *v/v*). The mixture was then incubated at room temperature for 30 min. Finally, the OD value of the colored product was measured at 595 nm against the reagent blank using spectrophotometry. Standard BSA solution (0–500 μg/mL) was used to make a standard graph for determination of the membrane protein concentration (Settakorn et al., 2022).

### 2.16 Determination of bilirubin concentrations

Plasma total bilirubin and direct bilirubin (conjugated bilirubin) concentrations were determined using Automated Clin Chem Analyzer (Cobas 8000 Modular Series, Roche Diagnostics International AG, Rotkreuz, Switzerland) according to the manufacturer’s instructions (Koonyosying et al., 2020) at the Central Laboratory, Maharaj Nakorn Chiang Mai Hospital, Faculty of Medicine, Chiang Mai University, Chiang Mai, Thailand.

### 2.17 Measurements of kidney function indicators

Blood urea nitrogen (BUN), plasma creatinine (Cr), and estimated glomerulus filtration rate (eGFR) were measured using Automated Analyzer (Cobas 8000 Modular Series, Roche Diagnostics International AG, Rotkreuz, Switzerland) according to the manufacturer’s instructions (Koonyosying et al., 2020) at the Central Laboratory, Maharaj Nakorn Chiang Mai Hospital, Faculty of Medicine, Chiang Mai University, Chiang Mai, Thailand.

### 2.18 UHPLC/ESI−QTOF/MS analysis of plasma catechin and metabolites

Heparinized plasma were prepared for analysis of catechins and metabolites using UHPLC/ESI−QTOF/MS according to the method mentioned above in Section 2.5 (Hodgson et al., 2014).

### 2.19 Statistical analysis

Data were analyzed using IBM SPSS Statistics 22 program and expressed as values of mean ± standard deviation (SD). Statistical significance was analyzed using one−way analysis of variance (ANOVA) with *post hoc* Tukey−Kramer, for which *p <* 0.05 was considered significant. When data were not distributed normally, non−parametric tests were used to determine significance.

## 3 Results

### 3.1 EGCG profile from HPLC−DAD and free-radical scavenging activity in GTE tablet

**FIGURE 1:**
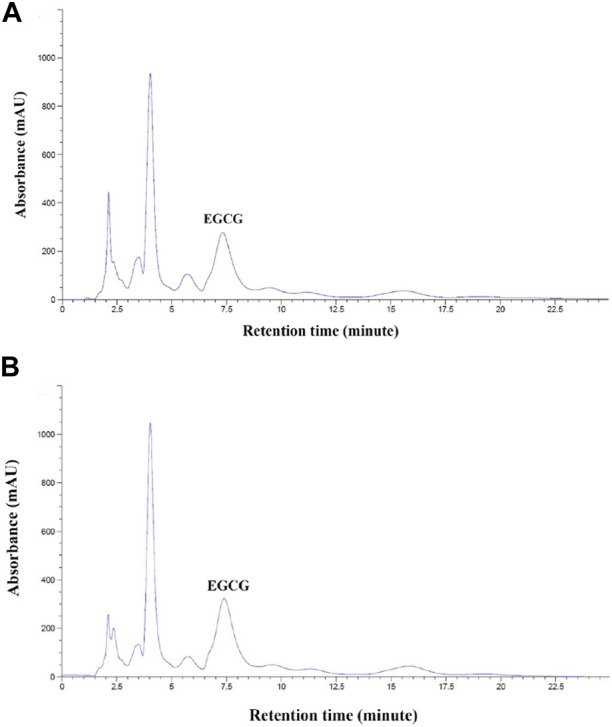
HPLC analysis of EGCG in GTE with concentrations of 10 mg/mL **(A)** and 50 mg/mL **(B)**. Abbreviations: EGCG = epigallocatechin−3−gallate, GTE = green tea extract, HPLC = high−performance liquid chromatography.

We found that EGCG in GTE was eluted at retention times in a range of 7.328–7.396 min (Figures 1A, B); thus, 1 g of crude GTE contained 55.48 ± 0.07 mg EGCG, while 750 mg of the GTE tablet was comprised of 7.86 ± 1.52 mg EGCG. As is shown in Table 1, the DPPH^•^−scavenging activity of the GTE tablet and Trolox showed half maximal inhibitory concentration (IC_50_) values of 0.1022 ± 0.0020 and 0.0220 ± 0.0019 mg/mL, respectively. In addition, the ABTS^•^−scavenging activity of the GTE tablet and Trolox showed IC_50_ values of 4.3560 ± 0.7940 and 0.6560 ± 0.0870 mg/mL, respectively.

**TABLE 1:** Inhibitory concentration values of GTE tablet and Trolox assayed using the DPPH and ABTS method. Data obtained from three independent experiments are expressed as mean ± SD values.

Method	Antioxidant	IC_50_ value (mg/mL)
DPPH	GTE tablet (50 mg EGCG eq)	0.102 ± 0.002
	Trolox	0.022 ± 0.002
ABTS	GTE tablet (50 mg EGCG eq)	4.356 ± 0.794
	Trolox	0.656 ± 0.087

Abbreviations: ABTS, 2,2'−azinobis−(3−ethylbenzothiazoline−6−sulphonic acid) diammonium salt, DPPH, 2,2−diphenyl−1−picrylhydrazyl, EGCG, eq = epigallocatechin−3−gallate equivalent, GTE, green tea extract and IC, inhibitory concentration.

### 3.2 Demographic data

Twenty−seven transfusion-dependent thalassemia (TDT) patients, including ten β−thalassemia major (BM), fifteen β−thalassemia/HbE (BE) (*n* = 15), and two with α/HbE Bart’s disease (AEB), were randomly divided into groups as follows: group 1 (T1−T8) receiving the placebo, group 2 (T9−T17) receiving the GTE tablet (50 mg EGCG equivalent, and group 3 (T18−T27) receiving green tea tablets (twice 50 mg EGCG equivalent). Individual and average information/numbers of genotypes, ages, Tx, iron chelator(s), splenectomy, and liver palpation, as well as the height and weight of the participants, are presented in Table 2. All of them (*n* = 27) were TDT patients who had been treated with different iron chelators, whereas the number of male and female subjects in groups 1 and 3, but not group 2, were distributed equally. In addition, the ages, height, BW, and liver span values were neither significantly different among these three groups.

**TABLE 2:** Demographic data (individual and mean ± SD values) of TDT patients at the time of enrollment. Patients were randomly divided into three groups: group 1 (T1−T8) received the placebo, group 2 (T9−T17) received GTE tablets (50 mg EGCG equivalent), and group 3 (T18−T27) received GTE tablets (100 mg EGCG equivalent).

Subject	Gender	Age (y)	Type	Tx	Chelation	Splen− ectomy	Height (cm)	BW (kg)	Liver span (cm)
T1	F	48	BE	yes	GPO−L1	no	156	47	17
T2	M	24	BM	yes	GPO−L1	yes	160	54	16
T3	F	38	BE	yes	GPO−L1	yes	145	46	10
T4	M	53	BE	yes	GPO−L1	no	160	53	15
T5	F	52	AEB	yes	GPO−L1	no	148	46	10
T6	M	38	BE	yes	GPO−L1	no	170	58	13
T7	F	46	BE	yes	GPO−L1	no	160	47	12
T8	M	29	BM	yes	GPO−L1	yes	160	43	12
Total	4F, 4M	43.4 ± 1.3	2BM, 5BE, 1H, 1AEB	8/1	9 GPO−L1	3/6	157.4 ± 7.8	49.2 ± 5.1	13.1 ± 2.6
T9	F	61	AEB	yes	GPO−L1	yes	135	30	10
T10	F	38	BE	yes	GPO−L1	yes	152	39	10
T11	M	33	BM	yes	GPO−L1	no	151	42	10
T12	M	34	BM	yes	GPO−L1+DFO	yes	150	44	12
T13	M	20	BE	yes	GPO−L1	yes	161	55	14
T14	F	26	BE	yes	GPO−L1	yes	158	48	13
T15	F	27	BM	yes	GPO−L1	yes	145	38	10
T16	F	23	BM	yes	GPO−L1	yes	156	40	10
T17	F	21	BM	yes	DFO + DFX	yes	145	45	10
Total	6F, 3M	31.4 ± 1.3	5BM, 3BE, 1AEB	9/0	7 GPO−L1, 1 GPO−L1+DFO, 1 DFO + DFX	8/1	150.3 ± 7.9	42.3 ± 7.0	11.0 ± 1.6
T18	F	31	BE	yes	GPO−L1	yes	150	46	13
T19	M	23	BE	yes	GPO−L1	yes	175	70	13
T20	F	19	BE	yes	GPO−L1	no	156	55	10
T21	F	35	BM	yes	DFO + DFX	yes	149	48	12
T22	M	28	BE	yes	GPO−L1	yes	175	54	10
T23	M	29	BE	yes	GPO−L1	no	166	50	10
T24	M	29	BE	yes	GPO−L1	yes	153	50	10
T25	M	46	BE	yes	GPO−L1	yes	175	58	18
T26	F	36	BM	yes	GPOL−1+DFO	no	165	60	13
T27	F	26	BM	yes	GPOL−1	yes	150	48	10
Total	5F, 5M	30.2 ± 7.5	3BM, 7BE	10/0	8 GPO−L1, 1 GPO−L1+DFX, 1 GPO−L1+DFO	7/3	161.4 ± 11.1	54.4 ± 7.2	11.9 ± 2.6

Abbreviations: AEB = α−thalassemia HbE Bart’s disease, BE = β−thalassemia/HbE, BM = β−thalassemia major**,** BW, body weight; DFO, deferrioxamine; DFP, deferiprone; DFX, deferasirox, GPO−L1 = generic deferiprone, F = female, M = male, Tx = transfusion.

### 3.3 RBC indices

**FIGURE 2:**
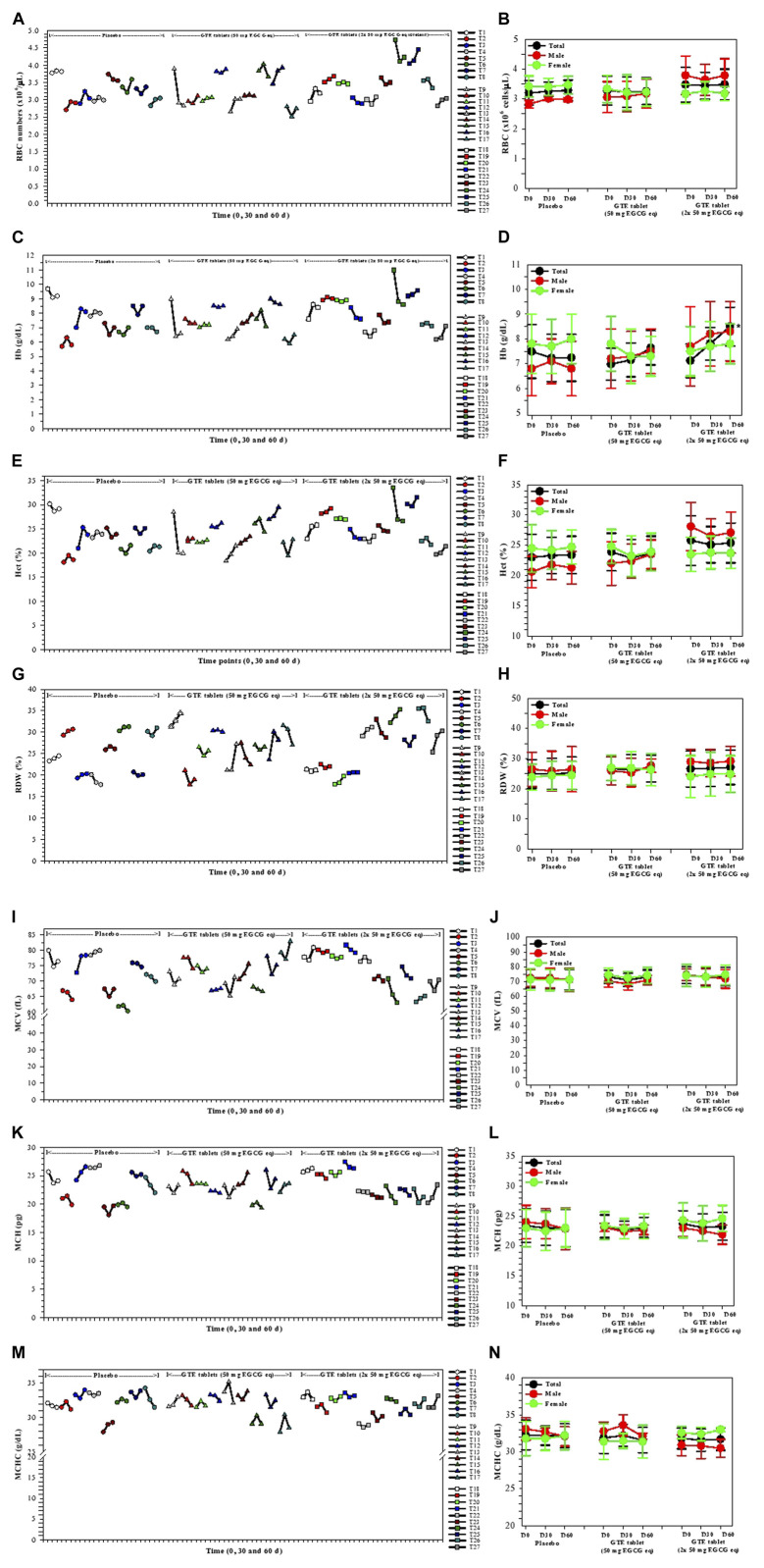
Changes in RBC index levels in the TDT patients who had consumed the placebo (T1−T8), GTE tablets (50 mg EGCG eq) (T9−T17), and GTE tablets (2 × 50 mg EGCG eq) (T18−T27) once daily for 60 days. Data have been presented as individual and mean ± SD values. Accordingly, *p < 0.05 when compared with day 0. Abbreviations: EGCG = epigallocatechin−3−gallate, EGCG eq = epigallocatechin−3−gallate equivalent, GTE = green tea extract, Hb = hemoglobin, Hct = hematocrit, MCH = mean corpuscular hemoglobin, MCHC = mean corpuscular hemoglobin concentration, MCV = mean corpuscular volume, RBC = red blood cells, RDW = red blood cell distribution width, TDT = transfusion−dependent β−thalassemia.

Values of RBC indices, including RBC numbers, Hb, Hct, MCV, MCH, MCHC, and RDW values, for subjects before and after they received treatments with the placebo or GTE tablets (50 mg and 2 × 50 mg EGCG equivalent) for 30 and 60 days are expressed in Figure 2. There were no changes of RBC numbers in all three study groups with regards to product treatment, treatment time or gender status (Figure 2B). Remarkably, Hb levels were increased tentatively in the group 2 subjects (*n* = 9) and significantly in the group 3 subjects (*n* = 10) on day 60 when compared with those on day 0; however, the effect was more distinct in the male subjects than the female subjects (Figure 2D). Alternatively, the GTE treatment, time course, and gender status did not influence the levels of the other RBC indices (Figures 2F, H, J, L, N, respectively). In addition, the individual changes in all of the parameters would indicate that the responders T2 and T8 (placebo); T11, T13, and T16 (GTE tablet: 50 mg EGCG eq); and T18, T19, T25, and T27 (GTE tablet: 2 × 50 mg EGCG eq) resulted in a continuous increase in RBC numbers during the course of the 60−day study (Figure 2A), which included T3; T13, T14, and T16; T18, T19, and T27 for Hb, Hct, and MCH values (Figures 2C,E,I, respectively); T1, T2, and T6; T9 and T13; T20, T22, T24, and T27 for RDW values (Figure 2M).

### 3.4 Plasma EPO, ERFE, and hepcidin concentrations

**FIGURE 3:**
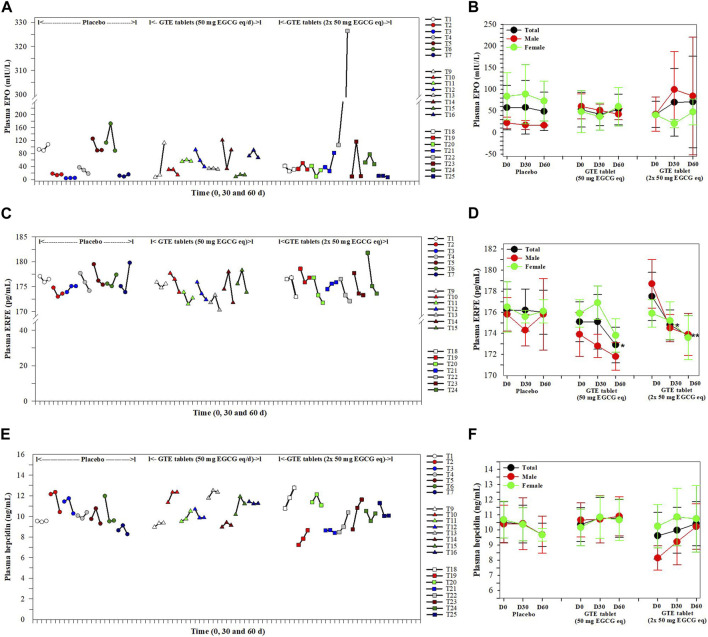
Levels of plasma EPO **(A, B)**, ERFE **(C, D)** and hepcidin **(E, F)** in TDT patients who had consumed the placebo (T1−T8), GTE tablets (50 mg EGCG equivalent) (T9−T17), and GTE tablets (2 × 50 mg EGCG equivalent) (T18−T27) once daily for 60 days. Data are presented in individual and mean ± SD values. Accordingly, *p < 0.05, **p < 0.01 when compared with placebo. Abbreviations: EGCG = epigallocatechin-3-gallate, EGCG eq = epigallocatechin−3−gallate equivalent, EPO = erythropoietin, ERFE = erythroferrone, GTE = green tea extract, TDT = transfusion-dependent thalassemia.

In the findings presented in Figure 3, levels of plasma EPO in the placebo and the two GTE tablet groups were not changed on days 30 and 60 when compared with those levels recorded at the beginning of the experiment and among these three groups (Figures 3A,B). Considerably, the plasma EPO concentrations in the T23 subjects increased continuously following consumption of the GTE tablets (2 × 50 mg EGCG eq/d) for 30 and 60 days. In contrast, plasma ERFE levels in GTE tablet (50 EGCG eq/d) group tended to be lower on day 30 and significantly reduced on day 60 (*p <* 0.05) when compared with day 0, while the levels of ERFE in GTE tablet group (100 mg EGCG eq/d) significantly decreased on days 30 (*p <* 0.05) and 60 (*p <* 0.01) when compared with day 0 (Figures 3C, D). Moreover, consumption of the GTE tablets (50 and 2 × 50 mg EGCG eq/d) daily for 30 and 60 days tended to increase levels of plasma hepcidin but not significantly when compared with day 0 (Figures 3E, F).

### 3.5 Inhibitory effect of AAPH−Induced RBC hemolysis

**FIGURE 4:**
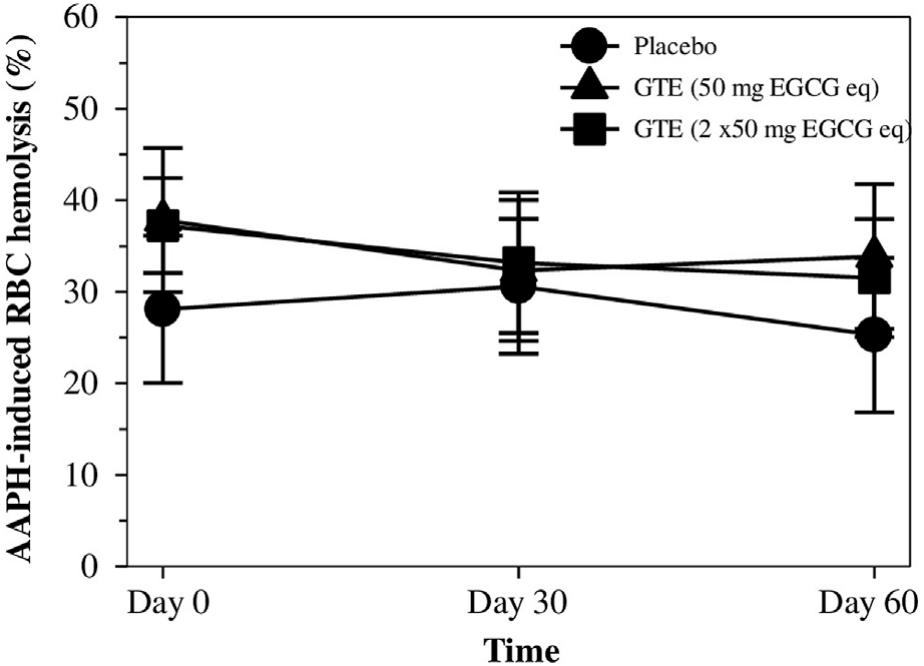
Percentage of hemolysis for RBC values of TDT patients who had consumed the placebo (T1-T8), GTE tablets (50 mg EGCG eq) (T9-T17), and GTE tablets (2 × 50 mg EGCG eq) (T18-T27) once daily for 60 days. Data are expressed as mean ± SD values. Abbreviations: 2,2′−Azo−bis(2−amidinopropane) dihydrochloride, EGCG = epigallocatechin−3−gallate, EGCG eq = epigallocatechin−3−gallate equivalent, GTE = green tea extract, RBC = red blood cells, TDT = transfusion-dependent thalassemia.

As is shown in Figure 4, the placebo did not protect against any degree of hemolysis of thalassemia subjects, while RBC was induced *ex vivo* by AAPH. Apparently, the percentage of RBC hemolysis tended to decrease in treatments of both GTE tablets (50 mg EGCG equivalent) and GTE tablets (100 mg EGCG equivalent) in a time−dependent manner, but neither decreases appeared to occur dose−dependently nor significantly when compared with subjects that had not undergone treatment.

### 3.6 RBC nonheme iron and TBARS contents in RBC membrane and plasma

**FIGURE 5:**
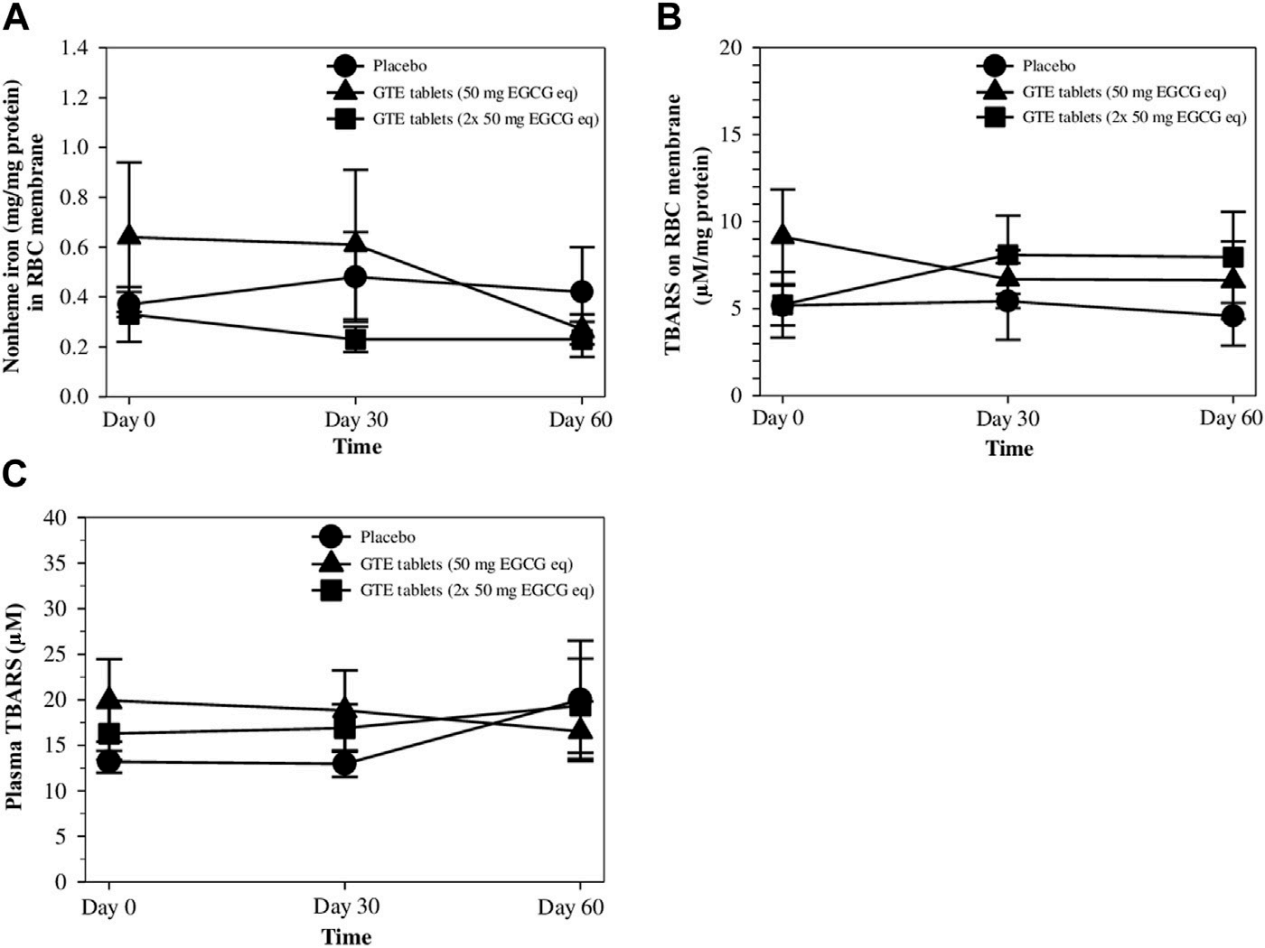
Levels of nonheme iron **(A)** and TBARS **(B)** in RBC membrane, and TBARS in the plasma **(C)** of TDT patients who had consumed the placebo (T1-T8), GTE tablets (50 mg EGCG equivalent) (T9-T17), and GTE tablets (2 × 50 mg EGCG equivalent) (T18-T27) once daily for 60 days. Data are expressed as mean ± SD values. Abbreviations: EGCG = epigallocatechin−3−gallate, EGCG eq = epigallocatechin−3−gallate equivalent, GTE = green tea extract, TBARS = thiobarbituric acid−reactive substances, TDT = transfusion-dependent thalassemia.

After nonheme iron concentrations were measured, the values were normalized according to the protein content of the RBC membrane, and the results are shown in Figure 5A. Interestingly, levels of nonheme iron in the RBC membranes tended to diminish after treatments with GTE tablets (50 and 100 mg EGCG equivalent), but non−significantly when compared with the placebo, wherein a 50 mg EGCG equivalent−dose seemed to be more effective. Similarly, measured TBARS contents in the RBC membrane and plasma were normalized according to their protein contents and the results are presented in Figures 5B, C, respectively. It was found that during 2 months of the study, TBARS levels in both GTE tablet groups and the placebo group were almost unchanged, while no significant differences were observed.

### 3.7 Plasma bilirubin concentrations

**FIGURE 6:**
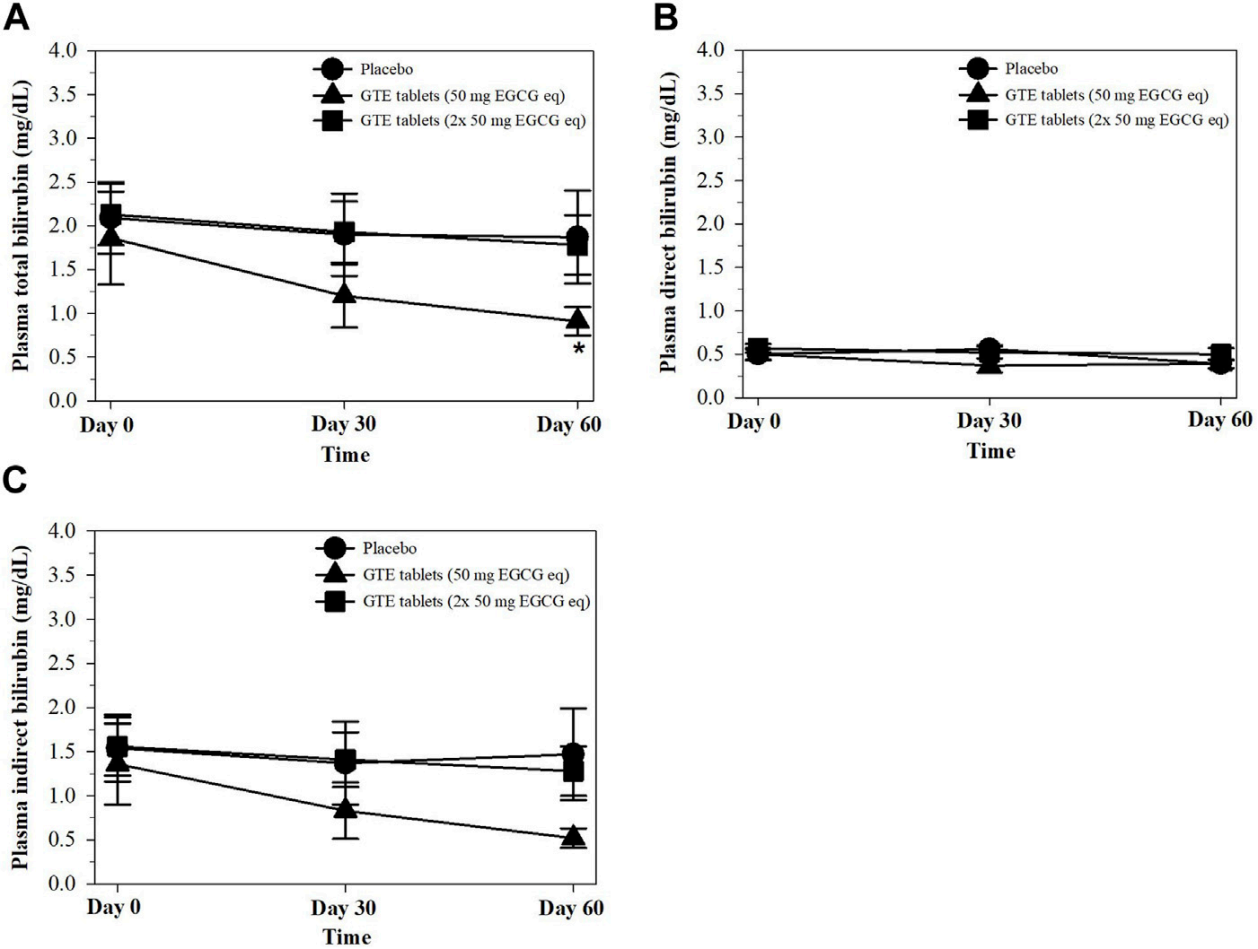
Levels of total bilirubin **(A)**, direct bilirubin **(B)**, and indirect bilirubin **(C)** in the plasma of TDT patients who had consumed the placebo or GTE tablets (50 and 100 mg EGCG eq) for 60 days. Data are expressed as mean ± SD values. Accordingly, *p < 0.05 when compared with the placebo and GTE tablet (100 mg EGCG eq). Abbreviations: EGCG = epigallocatechin−3−gallate, EGCG eq = epigallocatechin−3−gallate equivalent, GTE = green tea extract, TDT = transfusion-dependent thalassemia.

Physiologically, unconjugated (direct) bilirubin is a hydrophobic end−product obtained from heme degradation that must be conjugated with glucuronic acid by hepatic uridine diphosphate−glucuronyl transferase catalysis to form a conjugated (indirect) bilirubin and must be excreted via bile circulation. Pathologically, hyperbilirubinemia relates to an increase in RBC destruction, heme degradation, and liver dysfunctions. Herein, total and indirect bilirubin levels in the plasma were found to decrease in the GTE tablet (50 mg EGCG equivalent) group on days 30 and significantly on day 60 when compared with the placebo and GTE tablet (100 mg EGCG equivalent) groups (Figures 6A−C).

### 3.8 Kidney function parameters

As is shown in Table 3, levels of BUN, plasma Cr, and eGFR in the placebo and both GTE tablet groups did not change over 2 months of the study when compared with D0 levels and when comparisons were made between subjects who had or had not received GTE treatments. The findings could imply that consumption of GTE tablets had no influence on the kidney function of thalassemia patients.

**TABLE 3:** Levels of BUN, plasma Cr, and eGFR in TDT patients who had consumed the placebo (T1-T8), GTE tablets (50 mg EGCG eq) (T9-T17), and GTE tablets (2 × 50 mg EGCG eq) (T18-T27) once daily for 60 days. Data are expressed as mean ± SD values.

Treatment	Time	BUN (mg/dL)	Cr (mg/dL)	eGFR (mL/min/1.73 m^2^)
Placebo	D0	12.30 ± 0.72	0.50 ± 0.04	131.14 ± 4.14
	D30	13.12 ± 1.73	0.50 ± 0.04	132.82 ± 4.32
	D60	13.00 ± 1.16	0.49 ± 0.04	131.56 ± 3.95
GTE tablet (50 mg EGCG eq)	D0	12.80 ± 0.72	0.54 ± 0.03	138.02 ± 7.41
	D30	13.78 ± 2.64	0.50 ± 0.03	136.54 ± 6.46
	D60	11.90 ± 1.16	0.48 ± 0.03	130.73 ± 3.08
GTE tablet (2 × 50 mg EGCG eq)	D0	12.45 ± 0.69	0.51 ± 0.05	134.62 ± 5.04
	D30	13.75 ± 2.82	0.51 ± 0.05	136.92 ± 5.31
	D60	14.22 ± 1.22	0.53 ± 0.05	138.66 ± 5.04

Abbreviations: BUN, blood urea nitrogen; Cr, creatinine; EGCG, epigallocatechin−3−gallate, EGCG, eq = epigallocatechin−3−gallate equivalent, eGFR, estimated glomerulus filtration rate; GTE, green tea extract; TDT, transfusion-dependent thalassemia.

### 3.9 UHPLC−ESI−QTOF−MS assisted analysis of catechins in GTE tablet and plasma

**FIGURE 7:**
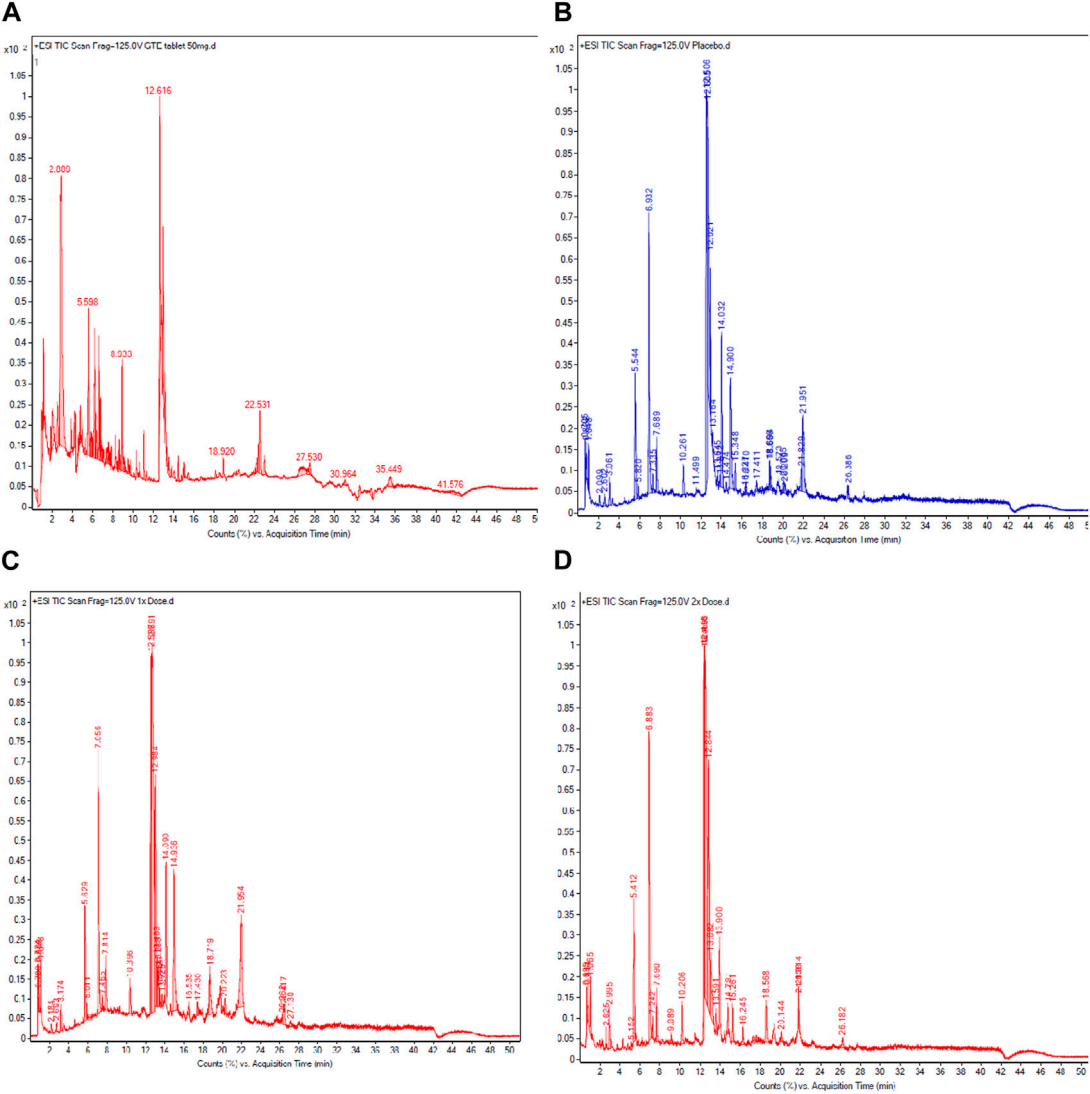
UHPLC−ESI−QTOF−MS profiles of catechins in GTE tablets **(A)**, catechins, and their metabolites in the plasma obtained from TDT patients who had consumed the placebo **(B)**, GTE tablets (50 mg EGCG equivalent) **(C)**, and GTE tablets (100 mg EGCG equivalent) **(D)** once daily for 60 days. Abbreviations: EGCG = epigallocatechin−3−gallate, GTE = green tea extract, TDT = transfusion-dependent thalassemia, TIC = total iron chromatogram, UHPLC−ESI−QTOF−MS = ultrahigh−performance liquid chromatography/electrospray ionization−quadrupole time−of−flight/mass spectrometry.

According to comprehensive UHPLC−ESI−QTOF−MS results (Figure 7; Table 4), the GTE tablets contained (-)-catechin (C), gallocatechin-(α->8)-epigallocatechin (GCEGC), epicatechin-2β->5,4β->6)-ent-epicatechin (ECeEC), gallocatechin or gallocatechin-4β-ol (GC), epicatechin 3-glucoside (ECGlu), 8-C-ascorbylepigallocatechin 3-gallate (AsEGCG), 4′,7-di-O-methylcatechin (DMC), epiafzelechin-(4β->8)-epicatechin 3,3′-digallate (EAECDG), epicatechin-(6'->8)-epicatechin (ECEC), catechin-(4α->8)-gallocatechin-(4α->8)-catechin (CGCC), (-)-epigallocatechin 7-glucuronide (EGCGluA), 3′,4′-methylenedioxy-5,7-dimethylepicatechin (MDDMEC), epigallocatechin-(4β->8)-epicatechin-3-O-gallate ester (EGCECG), robinetinidol-(4α->8)-catechin-(6->4α)-robinetinidol (RCR), epifisetinidol-(4β->8)-catechin (EFC), 4′-O-methyl-(-)-epicatechin-5-O-β-glucuronide (MEC5GluA), epigallocatechin 3-gallate (EGCG), epicatechin-(4α->8)-entepicatechin 3-gallate (ECECG), 8-C-ascorbylepigallocatechin 3-gallate (AsEGCG), gallocatechin or gallocatechin-4β-ol (GC), methylene bis-catechin (MBC), (-)-epigallocatechin 3-(4-methylgallate) (ECMG), 4β-(2-aminoethylthio)catechin (AETC), (-)-epigallocatechin 3-gallate 7-glucoside 4-glucuronide (EGCGGluGluA), gallocatechin-(α->8)-epigallocatechin (GCEGC), 4′-O-methyl-(-)-epicatechin-3′-O-β-glucuronide (4MEC3GluA), (-)-epigallocatechin 3-(4-methylgallate) (ECMG), 1,4′-methyl-(-)-epigallocatechin 3-(4-methylgallate) (MEGCMG), 3′-O-methyl-(-)-epicatechin 7-O-glucuronide (3MEC7GluA), 3-(4-hydroxybenzoyl)epicatechin (HBEC). Following consumption of placebo daily for 60 days, MECS, MEC3GluA, MC, epicatechin 3-glucoside (ECGlu), epicatechin-(4β->8)-gallocatechin (ECGC), MECGluA, ECEC, DMC, MBC, EFECEF and DGEC were detected in plasma from a representative subject. In comparison, MECS, MEC3GluA, MC, GC*, MEGCMG, 3′,4′-methylenedioxy-5,7-dimethylepicatechin (MDODMEC)*, ECGlu, ECGC, epicatechin-(6'->8)-epicatechin (ECEC)*, 3′-O-methyl-(-)-epicatechin 7-O-glucuronide (MECGluA), epicatechin-(6'->8)-epicatechin (ECEC), 3′,4′-methylenedioxy-5,7-dimethylepicatechin (MDDMEC)* and 4′,7-di-O-methylcatechin (DMC) in plasma from a representative subject who consumed GTE tablet (50 mg and 2 × 50 mg EGCG eq), in which the asterisk indicates the compounds that were detected only in the GTE tablet-treated sample.

**TABLE 4:** UHPLC−ESI−QTOF−MS analysis of catechins and metabolites detected in GTE tablets (50 mg EGCG eq) and the plasma of thalassemia patients in groups 1, 2, and 3 who had consumed the placebo and GTE tablets once daily for 60 days.

T_R_ (min)	Reference mass (g/mole)	Observed mass (m/z)	Chemical formula	Possible compounds	GTE tablet	Group 1	Group 2	Group 3
0.83	352.06	352.06	C_16_H_16_O_7_S	MECS		√		√
1.23	290.08	291.08	C_15_H_14_O_6_	C	√			
1.34	610.13	611.14	C_30_H_26_O_14_	GCEGC	√			
1.41	576.12	577.13	C_30_H_24_O_12_	ECeEC	√			
1.46	322.07	323.08	C_15_H_14_O8	GC	√			
1.64	452.13	470.17	C_21_H_24_O_11_	ECGlu	√			
1.79	632.10	650.14	C_28_H_24_O_17_	AsEGCG	√			
2.26	318.11	319.10	C_17_H_18_O_6_	DMC	√			
2.56	866.17	884.21	C_44_H_34_O_19_	EAECDG	√			
3.26	494.142	494.14	C_23_H_26_O_12_	MEC3GluA		√	√	√
3.49	578.14	579.15	C_30_H_26_O_12_	ECEC	√			
3.55	882.20	883.21	C_45_H_38_O_19_	CGCC	√			
3.60	482.10	505.09	C_21_H_22_O_13_	EGCGluA	√			
3.64	330.11	348.14	C_18_H_18_O_6_	MDDMEC	√			
3.71	746.14	747.16	C_37_H_30_O_17_	EGCECG	√			
3.86	866.20	867.21	C_45_H_38_O_18_	RCR	√			
3.97	562.14	580.18	C_30_H_26_O_11_	EFC	√			
4.14	480.12	503.12	C_22_H_24_O_12_	MEC5GluA	√			
4.39	458.08	481.07	C_22_H_18_O_11_	EGCG	√			
4.45	730.15	731.16	C_37_H_30_O_16_	ECECG	√			
4.91	632.11	650.14	C_28_H_24_O_17_	AsEGCG	√			
5.25	304.09	304.10	C_16_H_16_O_6_	MC		√	√	√
5.32	306.07	306.07	C_15_H_14_O_7_	GC	√		√	√
5.43	592.16	610.20	C_31_H_28_O_12_	MBC	√			
5.47	472.10	495.09	C_23_H_20_O_11_	ECMG	√			
5.78	365.10	383.13	C_17_H_19_NO_6_S	AETC	√			
5.95	796.17	797.18	C_34_H_36_O_22_	EGCGGluGluA	√			
6.01	442.09	443.10	C_22_H_18_O_10_	GC	√			
6.31	610.13	633.12	C_30_H_26_O_14_	GCEGC	√			
6.72	494.14	517.13	C_23_H_26_O_12_	4MEC3GluA	√			
6.92	456.10	457.11	C_23_H_20_O_10_	ECMG	√			
6.94	486.12	487.12	C_24_H_22_O_11_	MEGCMG	√		√	
7.52	478.15	501.14	C_23_H_26_O_11_	3MEC7GluA	√			
7.87	410.10	428.14	C_22_H_18_ O_8_	HBEC	√			
8.47	330.11	330.11	C_18_H_18_O_6_	MDODMEC			√	
10.60	452.13	452.13	C_21_H_24_O_11_	ECGlu		√	√	
14.09	594.14	594.14	C_30_H_26_O_13_	ECGC		√	√	
14.18	730.15	730.16	C_37_H_30_O_16_	ECEC			√	
14.87	494.14	494.14	C_23_H_26_O_12_	MECGluA		√	√	
18.27	576.12	576.13	C_30_H_24_O_12_	ECEC		√	√	
18.99	330.11	330.11	C_18_H_18_O_6_	MDDMEC				√
19.55	318.11	318.11	C_17_H_18_O_6_	DMC		√	√	√
36.10	592.16	592.16	C_31_H_28_O_12_	MBC		√		
36.70	834.22	834.22	C_45_H_38_O_16_	EFECEF		√		
37.40	594.10	594.10	C_29_H_22_O_14_	DGEC		√		

Abbreviations; AETC, 4β-(2-aminoethylthio)catechin, AsEGCG, 8-C-ascorbylepigallocatechin 3-gallate, C = (-)-catechin, CGCC, catechin-(4α->8)-gallocatechin-(4α->8)-catechin, DGEC, 3,5-digalloylepicatechin, DMC, 4′,7-di-O-methylcatechin, EAECDG, epiafzelechin-(4β->8)-epicatechin 3,3′-digallate, ECeEC, epicatechin-2β->5,4β->6)-ent-epicatechin, ECEC, epicatechin-(6'->8)-epicatechin, ECECG, epicatechin-(4α->8)-entepicatechin 3-gallate, ECGC, epicatechin-(4β->8)-gallocatechin, ECGlu, epicatechin 3-glucoside, ECGluA = epicatechin 3-glucoside, ECMG = (-)-epigallocatechin 3-(4-methylgallate), EFC, epifisetinidol-(4β->8)-catechin, EFECEF, epifisetinidol-(4β->8)-epicatechin-(6->4β)-epifisetinidol, EGCECG, epigallocatechin-(4β->8)-epicatechin-3-O-gallate ester, EGCG, epigallocatechin 3-gallate; EGCG, eq = epigallocatechin 3-gallate equivalent, EGCGluA = (-)-epigallocatechin 7-glucuronide, EGCGGluGluA = (-)-epigallocatechin 3-gallate 7-glucoside 4-glucuronide, GC, gallocatechin or gallocatechin-4β-ol, GCEGC, gallocatechin-(4α->8)α-epigallocatechin, GCEGC, gallocatechin-(α->8)-epigallocatechin, GTE, green tea extract; HBEC, 3-(4-hydroxybenzoyl)epicatechin, MDDMEC, 3′,4′-methylenedioxy-5, 7-dimethylepicatechin; MDODMEC, 3′,4′-methylenedioxy-5, 7-dimethylepicatechin, 3MEC7GluA = 3′-O-methyl-(-)-epicatechin 7-O-glucuronide, 4MEC3GluA = 4′-O-methyl-(-)-epicatechin-3′-O-β-glucuronide, MECS, 3′-O-methyl-(-)-epicatechin-5-O-sulphate, MC, 4′-O-methylcatechin, MBC, 8,8′-methylene bis-catechin, MEC3GluA = 4′-O-methyl-(-)-epicatechin-3′-Oβ-glucuronide, MEC5GluA = 4′-O-methyl-(-)-epicatechin-5-O-β-glucuronide, MEGCMG, 1,4′-methyl-(-)-epigallocatechin 3-(4-methylgallate), RCR, robinetinidol-(4α->8)-catechin-(6->4α)-robinetinidol, T_R_, retention time, UHPLC−ESI−QTOF−MS, ultrahigh performance liquid chromatography-electrospray ionization-quadrupole time-of-flight-mass spectrometry. m/z = mass−to−charge ratio and √ = present.

## 4 Discussion

Green tea (*C. sinensis*) extracts contain polyphenolics that include catechin derivatives; particularly EGCG, which exhibit a range of attractive effects and health benefits. We have previously reported on the properties for chelating redox active iron (e.g., nontransferrin bound iron and labile plasma iron in plasma and intracellular labile iron), the ability to inhibit lipid peroxidation, and the suppression of ERFE gene expression and production by GTE treatment, as well as the via consumption of concentrated GTE−curcuminoid (*Curcuma longa*) drinks, as can be seen in the cell cultures of β−knockout thalassemic mice and β−thalassemia patients with iron overload (Thephinlap et al., 2007; Upanan et al., 2015; Koonyosying et al., 2018; Koonyosying et al., 2019; Koonyosying et al., 2020; Settakorn et al., 2022). Gallocatechins such as EGCG, EGC, CGCC, EGCECG, ECECG, GC and GCEGC are naturally found in many plants and function to scavenge ABTS, membrane lipid peroxide and protein radicals more efficiently than catechins (Yang et al., 1999; Plumb et al., 2002; Hagerman et al., 2003). One UHPLC/QTOF/MS analysis elucidated 1802 metabolites in which 111 compounds were structurally identified as catechins, dimeric catechins, flavonol, flavone glycosides in green tea (*C. sinensis*) (Fuding Dabaicha leaves) (Dai et al., 2017). Another UHPLC/QTOF/MS identification of Chinese green tea has recently revealed 25 secondary metabolites in including flavanols, alkaloids, and polyphenols, and flavanol glycosides (Wang et al., 2021).

Herein, the GTE tablets used in this study contained different amounts of catechins, whereas the placebo contained nearly all the ingredients except for GTE powder. After thalassemia subjects had consumed the GTE tablets, most catechin constituents were biotransformed by the liver and new products. By using UHPLC/QTOF/MS, we have identified 31 phenolic compounds in our GTE as shown in this study, which a few compounds are the same and most of them are different. After ingestion, they are degraded by salivary catechin esterase and intestinal microbiota to small phenolic degradation products detectable in saliva, plasma and urine (van’t Slot and Humpf, 2009). Though EC, ECG, EGC and EGCG are four main polyphenolic ingredients in green tea, only EGC and EC instead of EGCG and ECG are found in blood circulation after ingestion of GTE product (Zhang et al., 1997). Importantly, EGCG can increase the proliferation and improve the survival of erythroid progenitors, suggestion a protection of erythropoiesis (Monzen and Kashiwakura, 2012). In biotransformation, catechin and its derivatives are metabolized by methylation, sulfation or glucuronidation in the liver and excreted in urine or/and feces via bile (Hackett and Griffiths, 1983b), also present a parent catechin and its metabolite 3′-O-methylcatechin (3′MC) in plasma (Donovan et al., 1999). After oral administration, DMC and MEC5GluA can conjugated with glucuronic acid (GluA) to form MEC3GluA and excreted both in bile, feces and urine (Hackett and Griffiths, 1981; Hackett and Griffiths, 1983a), consistently found in subjects’ plasma of this study. Possibly, the catechin metabolites which are present in plasma would be more biologically active than the forms existing in foods, giving protective nutrient (Donovan et al., 1999). Importantly, gallation (G) and B-ring dihydroxylation of C molecule will form EC, ECG, EGC, EGCG and other catechin gallates that vary in biological activities, accelerate the transfer of the catechin derivatives from the upper gastrointestinal tract to the small intestine, but delay subsequent transfers from the small intestine through the liver to plasma, from plasma to extravascular tissues and from kidneys to urine (Hodges et al., 2023). Consistently, half−maximal inhibitory concentration (IC_50_) values of ABTS^•^ and DPPH^•^ production by GTE tablets were found to be higher than in the other GT formulations (Al-Basher, 2019).

Ineffective erythropoiesis, inherited anemia, and secondary iron overload are the main characterizations in NTDT and TDT patients with so−called iron−loading anemia. Accordingly, occasional and/or multiple blood transfusions are given to these patients to maintain normal blood Hb levels, while iron can gradually accumulate in several vital organs in the body, leading to death of oxidative iron−induced cardiac arrythmia. Additionally, HbF−enhancing compounds (e.g., hydroxyurea) are used to increase Hb levels in HbE/β−thalassemia and NTDT patients; however, these compounds are very toxic (Algiraigri et al., 2017; Algiraigri and Kassam, 2017). Likewise, monotherapy of iron chelators and the relevant combinations were applied parenterally and/or orally to remove excessive iron depositions in tissues to prevent oxidative tissue damage and organ dysfunction. Therapeutic hepcidin mimetics (e.g., rusfertide) and hepcidin agonists (e.g., TMPRSS6 inhibitor and mini−hepcidin PR73 and mHS17) aim to reverse iron deficiency by targeting hepcidin−ferroportin axis, increase iron influx, mobilize tissue iron, and consequently normalize hematological parameters (Chua et al., 2015; Makis et al., 2021; Handa et al., 2023). Furthermore, oral ferroportin inhibitors (e.g., vamifeport and substituted benzoimidazole compounds) can relieve ineffective erythropoiesis and improve body iron parameters in iron overload−associated TDT mice, as well as treat patients suffering from neurodegenerative and cardiac diseases (Kadam et al., 2021; Kalleda et al., 2023). Previous studies have revealed that polyphenols dose dependently inhibit RBC hemolysis induced by AAPH, possibly by membrane anti-lipid peroxidation and protein antioxidation; EGCG and ECG had higher antioxidant capacity, while EGC and EC showed more effective ROS-radical scavenging activity (Zhang et al., 1997; Zou et al., 2001). Unfortunately, crude GTE, EGC and EGCG significantly decreased reduced glutathione (GSH) content, increased oxidized glutathione (GSSG) and methemoglobin levels in glucose-6-phosphate dehydrogenase-deficient RBC in a concentration-dependent manner, but not in normal RBC, suggesting a pro-oxidative effect by some of the catechins (Ko et al., 2006). Consistently, one study supports C, EC, EGCG, EGC and ECG show a strong inhibition of RBC hemolysis induced by oxidants (Grzesik et al., 2018). Surprisingly, ECGlu metabolite expressed hemopoietic effect by increasing RBC numbers and Hb content in male ICR rats treated with *Polygoni multiflori* Radix Praeparata product (Wang et al., 2020).

With regard to erythropoiesis, levels of RBC indices in TDT subjects were not changed by oral administration of GTE (50 and 100 mg EGCG equivalent/d) for 2 months, while plasma ERFE levels tended to decrease when compared with the baseline levels and in comparisons made between GTE treatment and the placebo groups. This finding revealed that Hb levels, Hct, and MCV values in the GTE−treated groups (1X and 2X dose) slightly increased when compared with the placebo group, even though these increases were not significant. Furthermore, RDW levels in the GTE−treated groups (1X and 2X dose) slightly decreased when compared with the placebo group. The GTE tablet induced small changes in hematopoietic activity parameters during the whole course of consumption when compared with the placebo group. Remarkably, Hb, Hct, and MCV values tended to increase in GTE−treated groups, which probably improved ineffective erythropoiesis. Moreover, RDW levels in the GTE−treated groups tended to decrease, which likely affected erythropoiesis and the RBC lifespan. Al-Momen and others reported that Hb levels were slightly increased in green tea−consuming thalassemia intermedia patients (Al-Momen et al., 2020). Beneficially, GTE consumption improved ineffective erythropoiesis and extended the lifespan of RBCs.

During erythropoiesis, EPO is required during the process, which is also essential for the treatment of anemia (Amer et al., 2010). Previous studies indicated that the levels of serum EPO increased in β−thalassemia patients to compensate for anemia (Nisli et al., 1997; Chaisiripoomkere et al., 1999). Therefore, we would like to investigate the effects of GTE to EPO. The present evidence has disclosed that plasma EPO in the GTE−treated groups (50 mg EGCG equivalent and 100 mg EGCG equivalent) were lower than in the placebo group. It could have possibly resulted from the improvement of RBC parameters (increasing the levels of Hb and Hct) after the GTE treatment. Casu C. and others confirmed that transmembrane protease serine 6 antisense oligonucleotide (TMPRSS6-ASO) is effective in ameliorating ineffective erythropoiesis through the induction of iron restriction. As a result, anemia was corrected, resulting in reduced levels of EPO (Casu et al., 2020). Erythroferrone (ERFE) is a new erythroid regulator that is secreted by erythroblasts in response to EPO activation (Arezes et al., 2018; Arezes et al., 2020). Moreover, ERFE may repress hepcidin (HCD) synthesis; consequently, it can be involved in erythropoiesis and iron metabolism. This study has revealed that the levels of plasma ERFE were reduced in the GTE-treated groups (50 mg EGCG equivalent and 100 mg EGCG equivalent). Specifically, in the group receiving 100 mg of EGCG equivalent, the levels of ERFE were significantly diminished in D30 and D60. The declining levels of plasma ERFE may have resulted from a reduction of EPO. Interestingly, treatments of green tea extract (GTE) and/or (DFP) could decrease levels of EPO and ERFE mRNA expression in the kidneys and spleen. The protein production in the plasma was also significantly decreased in iron-loaded β-globin gene knockout (BKO) mice, which led to decreases in the levels of plasma ferritin and iron contents in the liver, spleen, and kidney tissues (Settakorn et al., 2022). Previous studies have reported no correlations in serum ERFE levels with serum levels of ferritin, hepcidin, sTfR, EPO and GDF15, along with Hb and LIC in β-thalassemia intermedia patients (Schotten et al., 2017; Huang et al., 2019). Nevertheless, EPO activity has recently been reported to have a positive correlation with ERFE and GDF15 concentrations, but no correlations were observed between ERFE and hepcidin concentrations (Ozturk et al., 2021). In addition, a current study has demonstrated that blood transfusion could suppress EPO, ERFE, and soluble transferrin receptor (TfR) expressions but could decrease hepcidin production in TDT patients with β-thalassemia major (Zaman et al., 2023). Thus, ERFE can be acknowledged as a highly sensitive indicator of erythroid activity in β-TM. Herein, decreasing plasma ERFE levels, along with a tentative increase in the levels of plasma hepcidin in TDT patients, were predominantly observed in male subjects following the consumption of GTE tablets for 60 days. Taken together, the GTE treatment in TDT patients improved anemia by lowering thalassemic RBC hemolysis, as can be seen by a decrease in total and indirect bilirubin levels, along with an increase in the Hb levels. Subsequently the levels of the erythropoietic regulator hormones, including EPO and ERFE were lower, while levels of the systemic iron regulator, such as hepcidin, were increased.

According to iron-chelating property, EGCG treatment decreased iron deposition, transferrin (Tf) and transferrin receptor-1 (TfR1) protein expression, increased hepcidin mRNA level in the liver of alcoholic C57BL/6J mice (Ren et al., 2011). A strong antioxidant metabolite, 3′,4'−methylenedioxy−5,7−dimethylepicatechin was detected in our GTE tablet and ethanolic extract of rowanberry pomace (Sarv et al., 2023). In β−thalassemia patients, their RBC were accumulated with unpaired α−globin chains, resulting in imbalance occurred between α and β−globin chains and oxidative stress (Scott et al., 1993). This finding indicated that the consumption of GTE tablets could ameliorate oxidative stress. Since GTE contains polyphenolic compounds that manifest anti−oxidative activities, it can neutralize free radicals and may prevent the impairment occurring from free radical damage (Mandel et al., 2005; Nugala et al., 2012). Moreover, thalassemia RBC have unusual deposits of nonheme iron on membranes. This accelerates RBC injuries because iron can increase the generation of ROS (Repka et al., 1993; Browne et al., 1997). Furthermore, the present study indicated that levels of non-heme iron, RBC hemolysis, plasma total and indirect bilirubin tended to decrease after the GTE treatment but not significantly. In function, GTE displayed antioxidant, radical-scavenging and iron-chelating activities to protect the erythroid membrane from hemolysis (Costa et al., 2009; Saewong et al., 2010; Audomkasok et al., 2014). This defect can cause a decreased survival rate of RBCs by increasing RBC hemolysis. However, intervention with GTE tablets reduced the levels of hemolysis due to their antioxidant activities. We also found that total and indirect (unconjugated) bilirubin levels tended to be reduced after taking GTE tablets; thus, they would be good markers that is associated with an increase in RBC destruction (Barcellini and Fattizzo, 2015). Nevertheless, we have previously reported that levels of serum total bilirubin, direct bilirubin and total antioxidant capacity were not changes among TDT patients who consumed placebo or green tea and curcumin extract drink for 60 days (Koonyosying et al., 2020). This was possibly be due to their defensive response to iron−loaded oxidative stress (Bazvand et al., 2011). Taken together, GTE tablets exhibiting ROS-scavenging and iron-chelating properties could decrease oxidative stress and prolong RBC lifetime in β−thalassemia patients by removing redox-active nonheme iron, increasing antioxidant capacity, and inhibiting lipid peroxidation on the RBC membrane and plasma compartment.

Many publications have revealed that renal dysfunction is one of the complications in β−thalassemia patients due to inappropriate use of iron chelation, nephrotoxic drugs, and various infectious agents (Quinn et al., 2011; Musallam and Taher, 2012; Demosthenous et al., 2019). Alternatively, we must be concerned with the fact that natural products can cause nephrotoxicity (Nauffal and Gabardi, 2016). In our study, GTE consumption can prevent increases in plasma BUN and creatinine. Furthermore, eGFR which acts as a key indicator of renal function, tended to be increased in GTE−treated groups. Similarly, other investigators have reported that green tea has nephroprotective effects (Ben Saad et al., 2019). In this study, we found that the β−thalassemic plasma after GTE tablet consumption contained (+)−gallocatechin (GC), which is one of the catechins in green tea, and (±)-3′,4'−methylenedioxy−5,7−dimethylepicatechin. The metabolite forms of catechins include glucuronide, sulfate conjugates, or methylated conjugates (Chow and Hakim, 2011). This evidence indicates that (±)−3′,4'−methylenedioxy 5,7−dimethylepicatechin is an important new highlight associated with GTE−treated β−thalassemia plasma.

In terms of the possible limitations of the study, doses of EGCG in GTE tablets could not be scaled up since sizes of the GTE tablets would be bigger concordantly. The intervention time in this study was only 2 months, which may not have been long enough to show discriminant and significant changes in the RBC index and erythropoiesis parameter levels. Most importantly, all the parameter changes may be unclear in TDT patients who mainly depend upon regular transfusions of exogenous RBC, while the changes would be considerable in NTDT patients who are dependent upon their own erythropoietic activity along with a several−fold increase of duodenal iron absorption.

## 5 Conclusion

GTE tablets contain catechin derivatives, particularly EGCG, which are enzymatically biotransformed to many metabolites via the liver of thalassemia patients. Potentially, the catechins and their metabolites could increase Hb levels and decrease plasma ERFE concentrations, which were more predominant among male TDT patients than among female patients. Likewise, the compounds tended to increase plasma hepcidin, but decrease nonheme iron on the erythrocyte membrane. Collectively, consumption of GTE tablets could help prolong the survival of thalassemia RBC and decrease erythroferrone production, while consequently improving anemia and decreasing duodenal iron influx modulated via increased hepcidin production in thalassemia patients. In further research work, clinical studies should involve a larger study population, while the amount of time prescribed for the consumption of GTE tablets would need to be longer by up to 6–12 months.

## Figures and Tables

**Table:** 

**AA**	L−ascorbic acid
**AAPH**	2,2′−azo−bis(2−amidinopropane) dihydrochloride
**ABTS**	2,2'−azinobis−(3−ethylbenzothiazoline−6−sulphonic acid) diammonium salt
**ABTS** ^**•+**^	ABTS cationic radical
**AETC**	4β-(2-aminoethylthio)catechin
**ANOVA**	one−way analysis of variance
**AsEGCG**	8-C-ascorbylepigallocatechin 3-gallate
**BHT**	butylated hydroxytoluene
**BMI**	body weight index
**BUN**	blood urea nitrogen
**C**	(-)-catechin
**CGCC**	catechin-(4α->8)-gallocatechin-(4α->8)-catechin
**Cr**	creatinine
**DFX**	deferasirox
**DFO**	desferrioxamine
**DFP**	deferiprone
**DGEC**	3,5-digalloylepicatechin
**DI**	deionized
**DMC**	4′,7-di-O-methylcatechin
**DPPH**	2,2−diphenyl−1−picrylhydrazyl
**EAECDG**	epiafzelechin-(4β->8)-epicatechin 3,3′-digallate
**EC**	epicatechin
**ECeEC**	epicatechin-2β->5,4β->6)-ent-epicatechin
**ECEC**	epicatechin-(6'->8)-epicatechin
**ECECG**	epicatechin-(4α->8)-entepicatechin 3-gallate
**ECG**	epicatechin−3−gallate
**ECGC**	epicatechin-(4β->8)-gallocatechin
**ECGlu**	epicatechin 3-glucoside
**ECGluA**	epicatechin 3-glucoside
**ECMG**	(-)-epigallocatechin 3-(4-methylgallate)
**EFC**	epifisetinidol-(4β->8)-catechin
**EDTA**	ethylenediaminetetraacetic acid
**EFECEF**	epifisetinidol-(4β->8)-epicatechin-(6->4β)-epifisetinidol
**EGCECG**	epigallocatechin-(4β->8)-epicatechin-3-O-gallate ester
**EGC**	epigallocatechin
**EGCG**	epigallocatechin−3−gallate
**EGCGluA**	(-)-epigallocatechin 7-glucuronide
**EGCGGluGluA**	(-)-epigallocatechin 3-gallate 7-glucoside 4-glucuronide
**eGFR**	estimated glomerular filtration rate
**ELISA**	enzyme−linked immunosorbent assay
**EPO**	erythropoietin
**ERFE**	erythroferrone
**FAS**	ferrous ammonium sulfate
**GC**	gallocatechin or gallocatechin-4β-ol
**GCEGC**	gallocatechin-(4α->8)α-epigallocatechin
**GCEGC**	gallocatechin-(α->8)-epigallocatechin
**GDF15**	growth differentiation factor 15
**GTE**	green tea extract
**Hb**	hemoglobin
**HBEC**	3-(4-hydroxybenzoyl)epicatechin
**HCD**	hepcidin
**Hct**	hematocrit
**IC**	inhibitory concentration
**HPLC−MS/MS**	high performance liquid chromatography−mass spectrometry
**LIC**	liver iron content
**MCC**	microcrystalline cellulose
**MCH**	mean corpuscular hemoglobin
**MCHC**	mean corpuscular hemoglobin concentration
**MCV**	mean corpuscular volume
**MDDMEC**	3′,4′-methylenedioxy-5,7-dimethylepicatechin
**MDODMEC**	3′,4′-methylenedioxy-5,7-dimethylepicatechin
**3MEC7GluA**	3′-O-methyl-(-)-epicatechin 7-O-glucuronide
**4MEC3GluA**	4′-O-methyl-(-)-epicatechin-3′-O-β-glucuronide
**MECS**	3′-O-methyl-(-)-epicatechin-5-O-sulphate
**MC**	4′-O-methylcatechin
**MBC**	8,8′-methylene bis-catechin
**MEC3GluA**	4′-O-methyl-(-)-epicatechin-3′-Oβ-glucuronide
**MEC5GluA**	4′-O-methyl-(-)-epicatechin-5-O-β-glucuronide
**MEGCMG**	1,4′-methyl-(-)-epigallocatechin 3-(4-methylgallate)
**MOPS**	4−morpholinepropanesulfonic acid
**m/z**	mass−to−charge ratio
**NSS**	normal saline solution
**NTBI**	non−transferrin bound iron
**NTDT**	non−transfusion−dependent thalassemia
**PBS**	phosphate buffered saline
**PVPK90**	polyvinylpyrrolidone K90
**RBC**	red blood cells
**RCR**	robinetinidol-(4α->8)-catechin-(6->4α)-robinetinidol
**RDW**	red blood cell distribution width
**ROS**	reactive oxygen species
**RP−HPLC**	reverse−phase high performance liquid chromatography
**SD**	standard deviation
**SI**	serum iron
**sTfR**	soluble transferrin receptor
**TBA**	thiobarbituric acid
**TBARS**	thiobarbituric acid−reactive substances
**TCA**	trichloroacetic acid
**TDT**	transfusion−dependent thalassemia
**TI**	thalassemia intermedia
**TIBC**	total iron−binding capacity
**TM**	thalassemia major
**TMPRSS6-ASO**	transmembrane protease serine 6 antisense oligonucleotide
**TR**	retention time
**Trolox**	6−hydroxy−2,5,7,8−tetramethylchroman−2−carboxylic acid
**TSAT**	transferrin saturation
**TT**	thalassemia trait
**UHPLC−ESI−QTOF−MS**	ultrahigh performance liquid chromatography-electrospray ionization-quadrupole time-of-flight-mass spectrometry
**√**	present

## Data Availability

The original contributions presented in the study are included in the article/Supplementary Material, further inquiries can be directed to the corresponding author.
