# Supplementary material for: A randomized placebo−controlled clinical trial of oral green tea epigallocatechin 3−gallate on erythropoiesis and oxidative stress in transfusion−dependent β−thalassemia patients
Source: Front Mol Biosci. 2024 Jan 24;10:1248742. doi: 10.3389/fmolb.2023.1248742 (PMC10848917; doi:10.3389/fmolb.2023.1248742)
Supplement: Supplementary file 1 [file DataSheet2.docx]

**CLINICAL TRIAL PROTOCOL**

**SPONSOR:** Faculty of Medicine, Chiang Mai University, Chiang Mai, Thailand

**PROTOCOL NAME:** Study effect of consumption of green tea extract tablets for amelioration of iron overload and oxidative stress in transfusion−dependent β−thalassemia patients

**Funders:** 1. Thailand Science Research and Innovation (TSRI) Academic Section

2. Faculty of Medicine Fund, Chiang Mai University

| PROTOCOL NO. | STUDY CODE: MED-2561-05846  Research ID: 05846 | | |
| --- | --- | --- | --- |
| VERSION NO. | 1 (Case Record Form edited 20 AUG 2019) | DATE | 22/05/2020 |
| PRINCIPLE INVESTIGATOR(S) | Associate Professor Adisak Tantiworawit, M.D. | | |
| RESEARCH TEAM MEMBERS | 1. Associate Professor Pimlak Charoenkwan, M.D.  2. Assistant Professor Sasinee Hantrakool, M.D.  3. Professor Somdet Srichairatanakool, Ph.D.  4. Assistant Professor Pimpisid Koonyosying, Ph.D.  5. Mr. Touchwin Petiwathayakorn, B.Sc.  6. Miss Kornvipa Settakorn, B.Sc. | | |

**APPROVED BY:**

|   (Associate Professor Nimit Morakote, Ph.D.)  Chairperson of Human Ethical Committee,  Faculty of Medicine, Chiang Mai University | Date 00/05/2020 |
| --- | --- |
| *Principle Investigator or Sponsor Signature and Title* |  |


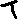

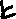

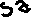

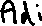


| Associate Professor Adisak Tantiwoarawit, M.D.  Principal Investigator Date 01/07/2020 |
| --- |
| SITE: Adult Thalassemia Clinic (OPD 23), 11^th^ Floor, Sriphat Building, Maharaj Nakorn Chiang Mai Hospital, Faculty of Medicine, Chiang Mai University, Chiang Mai, Thailand. |

# PROTOCOL AGREEMENT

I have read and understand the protocol below. In my capacity as Investigator, my duties include making sure of the safety of the study participants enrolled under by supervision and providing [PI or SPONSOR NAME] with complete and timely information. This information will be provided as outlined in this study protocol. All the information relating to this study will be held in strict confidence and this confidentiality requirements applies to all staff at this study site or involved with this study. I agree to maintain the procedures required to perform this study in accordance with Good Clinical Practice principles and to abide by the terms of this protocol.

| PROTOCOL NO. | STUDY CODE: MED-2561-05846  Research ID: 05846 | PROTOCOL DATE | 22/05/2020 |
| --- | --- | --- | --- |
| PROTOCOL TITLE | 1 (Case Record Form edited 20 AUG 2019) | | |

| 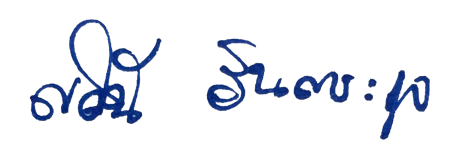 |  |
| --- | --- |
| *Investigator Signature* | Date 22/05/2020 |

Sasinee Hantrakool, M.D.

|  |
| --- |
| SITE: Adult Thalassemia Clinic, Division of Hematology, Department of Internal Medicine, Faculty of Medicine, Chiang Mai University, Chiang Mai, Thailand. |

####

| CONTACT INFORMATION | Office: Division of Hematology, Department of Internal Medicine, Faculty of Medicine, Chiang Mai University, Chiang Mai 50200, Thailand  Mobile phone number: 66-0813243280  Email: [sasinee.h@cmu.ac.th](mailto:sasinee.h@cmu.ac.th) |
| --- | --- |

####

Table of Contents

[PROTOCOL AGREEMENT 2](#_Toc8822337)

[1. ABSTRACT 4](#_Toc8822338)

[2. BACKGROUND AND SIGNIFICANCE OF PRELIMINARY STUDIES 4](#_Toc8822339)

[3. STUDY AIMS 5](#_Toc8822340)

[4. ADMINISTRATION 5](#_Toc8822341)

[5. STUDY DESIGN 5](#_Toc8822342)

[6. Study Procedures 6](#_Toc8822343)

[SUBJECT SELECTION PROCEDURES 6](#_Toc8822344)

[RANDOMIZATION PROCEDURES (if applicable) 6](#_Toc8822345)

[STUDY INTERVENTION 6](#_Toc8822346)

[7. SAFETY MONITORING PLAN 7](#_Toc8822347)

[8. ANALYSIS PLAN 8](#_Toc8822348)

[9. REFERENCES 8](#_Toc8822349)

# ABSTRACT

| β−Thalassemia patients suffer from ineffective erythropoiesis and increased red blood cell (RBC) hemolysis. Blood transfusion, erythropoietic enhancement, and antioxidant supplementation can ameliorate chronic anemia. Green tea extract (GTE) is comprised of catechin derivatives, of which epigallocatechin−3−gallate (EGCG) is the most abundant, presenting free−radical scavenging, iron−chelating, and erythropoiesis−protective effects. The present study aimed to evaluate the effects of GTE tablets on the primary outcome of erythropoiesis and oxidative stress parameters in transfusion−dependent β−thalassemia (TDT) patients. Twenty−eight TDT patients were randomly divided into placebo and GTE tablet (50 and 100 mg EGCG equivalent) groups and assigned to consume the product once daily for 60 d. Blood was collected for analysis of hematological, biochemical, and oxidative stress parameters. Accordingly, consumption of GTE tablets did not improve RBC indices when compared with the placebo; however, there were more responders to the GTE tablets. Interestingly, amounts of nonheme iron and thiobarbituric acid−reactive substances in RBC membranes tended to decrease in both GTE tablet groups when compared with the placebo. Importantly, consumption of GTE tablets lowered plasma levels of erythroferrone (*p <* 0.05) and reduced bilirubin non−significantly and dose−independently. Thus, GTE tablets could improve RBC hemolysis and modulate erythropoiesis regulators in transfusion−dependent thalassemia patients. |
| --- |

# BACKGROUND AND SIGNIFICANCE OF PRELIMINARY STUDIES

| Ineffective erythropoiesis (IE) in β−Thalassemia patients is genetically caused by the absence or diminished production of β−globin chains, leading to an excess and precipitation of α−globin chains, as well as oxidative stress in erythroid precursors (Cao and Galanello 2010, Galanello and Origa 2010). In addition, reduced erythroid cell differentiation and survival, together with increased red blood cell (RBC) hemolysis, will worsen the anemia (Rivella 2019). A high increase of duodenal iron absorption is predominantly found in non transfusion−dependent β−thalassemia (NTDT) patients, while repeated blood transfusions are required for transfusion−dependent β−thalassemia (TDT) patients to compensate for the IE and maintain normal blood hemoglobin (Hb) levels (Rivella 2012, Taher, Weatherall et al. 2018). Consequently, secondary iron overload induces oxidative tissue damage and escalates the mortality in these patients (Taher and Saliba 2017). Nowadays, iron chelators, such as desferrioxamine (DFO), deferiprone (DFP), and deferasirox (DFX), are being used for the treatment of β−thalassemia patients with iron overload, even when showing adverse effects (Viprakasit and Origa 2014, Cappellini and Motta 2017). Recently, novel therapeutics for β−thalassemia patients involving Janus kinase (Jak) inhibitors, hepcidin agonists, such as minihepcidin and transmembrane serine protease 6 (TMPRSS6) antagonist, and ferroportin inhibitors, such as VIT-2763 and apo−transferrin, have been reported to improve iron dysregulation (Rivella 2012, Gelderman, Baek et al. 2015, Makis, Voskaridou et al. 2021). Interestingly, allogeneic hematopoietic stem cell transplantation involving gene therapy with γ− or β−globin insertion, transformed growth factor−β ligand traps, such as luspatercept, pyruvate kinase activators, such as mitapivat, and fetal hemoglobin (HbF) inducers, such as phosphodiesterase 9 inhibitor tovinontrine (IMR−687) and resveratrol, have been reported to restore normal erythropoiesis (Theodorou, Phylactides et al. 2016, Makis, Voskaridou et al. 2021, Matte, Federti et al. 2021). Erythropoietin (EPO) is synthesized by the kidneys to activate marrow erythrocytic progenitors for RBC synthesis and to respond to hypoxia (Amer, Dana et al. 2010). Orchestrally, erythroferrone (ERFE) is an erythroid regulator secreted by erythroblasts in response to EPO activation and the repression of hepcidin synthesis by the liver to mediate iron overload (Kautz and Nemeth 2014, Kautz, Jung et al. 2015). In NTDT patients, high amounts of ERFE from the expanded pool of immature erythroid cells can suppress hepcidin expression and production, and consequently exacerbate their ineffective erythropoiesis and anemia (Theodorou, Phylactides et al. 2016, Pagani, Nai et al. 2019).  Green tea (*Camellia sinensis*) extract (GTE) contains catechins, gallocatechin 3−gallate, (-)−epicatechin (EC), (-)−epigallocatechin (EGC), (-)−epicatechin−3−gallate (ECG), and (-)−epigallocatechin−3−gallate (EGCG) (Chacko, Thambi et al. 2010, Khan and Mukhtar 2013), which can be analyzed effectively using high−performance liquid chromatography equipped with a diode array detector (HPLC−DAD) in conjunction with electrospray ionization−mass spectrometry (HPLC/ESI−MS) and ultraHPLC/ESI−MS time of flight (UHPLC/ESI−TOF/MS) (Clarke, Dew et al. 2014, Dai, Xie et al. 2017, Koonyosying, Kongkarnka et al. 2018). Interestingly, EGCG, as the most abundant compound, possesses antioxidant, anticancer, radical−scavenging, anti−inflammatory, and cardiovascular−protective properties (Mandel, Avramovich-Tirosh et al. 2005). In addition, the compound can remove excessively−accumulated iron in tissues, redox−active iron in the plasma and RBC membrane (Thephinlap, Ounjaijean et al. 2007), and relieve hemolysis of thalassemia RBC (Saewong, Ounjaijean et al. 2010). Importantly, treatment of GTE (50 mg EGCG equivalent) lowered expressions of *Epo* mRNA in the kidneys and *Erfe* mRNA in the spleen, plasma EPO, ERFE, and ferritin concentrations, tissue iron content, and lipid-peroxidation products in the tissues and plasma of iron−loaded BKO mice (Settakorn, Kongkarnka et al. 2022). Thus, manipulations of iron metabolism, erythropoietic activity, or erythroid cell differentiation, and survival could improve iron overload and anemia in β−thalassemia patients. |
| --- |

# STUDY AIMS

| We hypothesized that green tea extract which is rich in EGCG and exerts antioxidative, free−radical scavenging and iron−chelating activities could enhance erythropoiesis, ameliorate oxidative RBC, and prolong RBC survival in β−thalassemia patients. The present study aimed to investigate whether consumption of GTE could effectively influence levels of erythropoiesis and oxidative stress parameters in TDT patients. |
| --- |

# ADMINISTRATION

Describe the participating study sites, units, laboratories, data centers, and any coordinating centers applicable.

| Participating study sites, units: Adult Thalassemia Clinic (OPD 23), 11^th^ Floor, Sriphat Building, Maharaj Nakorn Chiang Mai Hospital, Faculty of Medicine, Chiang Mai University, Chiang Mai, Thailand.  Laboratories:  1. Hematology Laboratory, Department of Internal Medicine, Faculty of Medicine, Chiang Mai University, Chiang Mai 50200, Thailand  2. Central Laboratory, Maharaj Nakorn Chiang Mai Hospital Faculty of Medicine, Chiang Mai University, Chiang Mai 50200, Thailand  3. Biochemistry Laboratory, Department of Biochemistry, Faculty of Medicine, Chiang Mai University, Chiang Mai 50200, Thailand  4. Drug Formulation Laboratory, Department of Pharmaceutical Sciences, Faculty of Pharmacy, Chiang Mai University, Chiang Mai 50200, Thailand  Data centers: Adult Thalassemia Clinic (OPD 23), 11^th^ Floor, Sriphat Building, Maharaj Nakorn Chiang Mai Hospital, Faculty of Medicine, Chiang Mai University, Chiang Mai, Thailand. |
| --- |

# STUDY DESIGN

Describe the study design, population, sample size, power analyses, anticipated outcomes, and endpoints.

| This study was conducted in accordance with the Declaration of Helsinki and approved by the Ethical Committee for Human Study of the Faculty of Medicine, Chiang Mai University, Chiang Mai, Thailand (Reference Number: MED-2561-05846; Date of approval: 22-05-2020). All participants gave their informed consent for their inclusion in this study. The clinical study protocol was reviewed and approved by the Thai Clinical Trials Registry (TCTR) committee of the Medical Research Foundation of Thailand (ID: TCTR20211118002; Date of Registration: 18/11/2021). This study was conducted in accordance with the reporting guidelines of the Consolidated Standards of Reporting Trials (CONSORT) 2010. Then, the study was conducted from 01/07/2020 – 31/05/2021 at the Adult Thalassemia Clinic, Maharaj Nakorn Chiang Mai Hospital, Faculty of Medicine, Chiang Mai University, Chiang Mai, Thailand. |
| --- |

# Study Procedures

## SUBJECT SELECTION PROCEDURES

| For the inclusion criteria, all subjects were Thai adult TDT patients aged 20−65 years, who could communicate in the Thai language, visited the clinic for regular treatments and who had not received extra-treatments for at least three months before and during the study. For the exclusion criteria, subjects are pregnant, lactating or unwilling to use the tested compounds/product. For the subject withdrawal criteria, subjects are unwilling to continue the study with themselves of according to a doctor’s comments, do not follow the study regulations/instruction. Accordingly, participants (n = 33) were recruited for the study, randomly divided into three groups of equal numbers (n = 11) and treated as will be explained below. Thai TDT patients aged 20−65 years old were enrolled in this study. Patients, the placebo group 1 (n = 8) and two intervention groups 2 and 3 that received either 50 mg EGCG equivalent−GTE (n = 9) or 100 mg EGCG equivalent−GTE (n = 10), respectively. The latter two groups were orally administered the GTE tablets 30 minutes before breakfast once a day for two months according to the same schedule with placebo group. This study was conducted from 01/07/2020 to 31/05/2021. Blood samples were analyzed for RBC indices, anti RBC hemolysis activity, plasma EPO, ERFE, bilirubin, urea nitrogen, creatinine and thiobarbituric acid−reactive substances (TBARS) concentrations, nonheme iron and TBARS contents in RBC membrane, and catechins and the metabolites in plasma. |
| --- |

## RANDOMIZATION PROCEDURES (if applicable)

| Not applicable |
| --- |

## STUDY INTERVENTION

| For drug or device studies, describe the Active study agent(s), Placebo study agents(s), Blinding, labeling, agent(s) preparation, Storage, Administration and Toxicities  **Active study agent: Green tea extract tablets**  Tea trees (*Camellia sinensis*) were cultivated for 3−4 years in a tea field located in Mon Ngao, Mae Tang District, Chiang Mai Province, Thailand and managed by the Royal Project Foundation (RPF) in Chiang Mai. Fresh tea shoots were collected by local tea pickers under the supervision of Miss Banthita Buamasang from the RPF. The shoots were then immediately transported to the Department of Biochemistry, Faculty of Medicine, Chiang Mai University in Chiang Mai for processing. GTE was prepared using the method established by Koonyosying and colleagues (Koonyosying, Kongkarnka et al. 2018). Briefly, tea shoots were immediately inactivated by an inherent polyphenol oxidase and dried at 200 °C for 3-5 minutes using a microwave oven (Electrolux^®^, Stockholm, Sweden, 20-L capacity, 4000-watts electric power). Dried tea shoots were ground using an electric blender (SharpThai Company, Limited, Thailand), extracted with hot deionized water (DI) (80 °C, 1 kg/10 L) for 10 min and then filtered through a membrane (cellulose acetate type, 0.45 µm pore size, GE Healthcare Life Sciences, Whatman, Maidstone, Kent, UK) under a vacuum(Upanan, Pangjit et al. 2015). Later, liquid GTE was mixed with maltodextrin (5%, *w*/*v*) and rapidly dried using a spray dryer (T.S.K. Engineering Company, Limited, Chonburi, Thailand) to transform the liquid into fine GTE particles. Finally, spray-dried GTE was packed into an aluminum foil bag (10 kg capacity) and kept at 4 °C in a refrigerator until being used. Afterwards, spray-dried GTE powder was mixed with microcrystalline cellulose (MCC) (2:1, *w*/*w*). Polyvinylpyrrolidone-K90 (PVP-K90) was dissolved in 95% ethyl alcohol (5%, *w/w*), which was then used as a binding solution and added into the GTE/MCC mixture gradually until a homogenous PVP-K90 solution was reached at a final concentration of 1% (*w/w*). The mixture was passed through a nylon net filter (size 14 mesh), and the granules were dried for 15 minutes using a fluid-bed dryer. Afterwards, dry GTE granules were compressed into an oval-shaped tablet (50 mg EGCG each) in a practical laboratory located at the Faculty of Pharmacy, Chiang Mai University, Chiang Mai, Thailand. Finally, a GTE tablet sample was randomly selected for quantification of EGCG using the HPLC system (Model 1290 Infinity II, Agilent Technologies, Inc., Santa Clara, CA, USA)(Upanan, Pangjit et al. 2015). First of all, spray-dried GTE powder (10 mg) was reconstituted in 1 mL of deionized water (DI) and filtered through a syringed membrane filter (polytetrafluoroethylene type, 0.45-μm pore diameter, 13 mm filter diameter, Monotaro Company, Limited, Tokyo, Japan). In our analysis, 20 µL of sample or standard EGCG (0.625-1 mg/mL) was loaded using an autosampler into the HPLC coupled with a diode array detector, fractionated onto a column (ODS2-type, 150 mm x 4.6 mm, 5 µm pore size, Agilent Technologies, Inc., Santa Clara, CA, USA), eluted with mobile-phase solvent of 0.05% H_2_SO_4_: acetonitrile: ethyl acetate (86:12:2, *v/v/v*) at a flow rate of 1.0 mL/min and the eluents were then detected at a wavelength of 280 nm. EGCG was identified using the same retention time (T_R_) as the EGCG standard. Its concentration was determined using a standard curve made from different concentrations of the EGCG standard.  Placebo study agent: Placebo tablets were comprised of all ingredients except GTE, and these had the same shape, size and color of the GTE tablets (Settakorn, Hantrakool et al. 2022, Settakorn, Kongkarnka et al. 2022).  Blinding, labeling, agent(s) preparation: We labelled “P” for placebo agent and “T” for active GTE” at the product bottom.  Storage: Placebos and GTE tablets were kept in white polypropylene bottles (30 tablets each) with sealed caps at 4 °C in a refrigerator until being used.  Administration: The placebo group (n = 11), 50 mg EGCG equivalent-GTE group (n = 9) and twice 50 mg EGCG equivalent-GTE group (n = 10) were orally administered 30 minutes before breakfast once a day for two months. Notably, all the participants were asked to avoid consuming foods that were rich in polyphenolic compounds.  Toxicities: Our previous studies have demonstrated safety for using GTE powder and product up to the dose of 100 mg EGCG equivalent in cells, rodents and humans. |
| --- |
| For other types of studies, describe the Intervention and Control group:  For the inclusion criteria, all subjects were Thai adult TDT patients aged 20-65 years, who could communicate in the Thai language, visited the clinic for regular treatments and who had not received extra-treatments for at least three months before and during the study. Thirty-three participants were recruited for the study. They were randomly divided into three groups of equal numbers (n = 11) and treated as will be explained below. However, three participants withdrew from the study for personal reasons. |

# SAFETY MONITORING PLAN

*For the safety monitoring plan, describe the:*

- *Adverse event and serious adverse event definitions.*
- *Subject safety procedures.*
- *How adverse events will be identified, documented, and reported.*
- *The review frequency of safety information summaries and review procedures.*
- *The safety and efficacy stopping rules.*

| Adverse event (AE) is any symptom and sign and abnormal laboratory values that a patient experiences after administration of a study agent.  Serious adverse event (SAE) is any untoward occurrence that is fatal or life-threatening, requires hospitalization or a prolonged hospitalization, leads to significant disability or incapacity, or results in a congenital anomaly.  Adverse event is monitor by the investigator during study enrollment and document in patient record and report to ethical committee (EC). SAE is report within 24 hours.   AE and SAE will be review as not relate, unlikely, possible, probable related to study agent by investigator and report to EC.  If the AE and SAE review and safety measures in term of safety to protect the subjects of a clinical trial against any immediate event and the study agent must be stopped as soon as the event is aware. |
| --- |

# ANALYSIS PLAN

| **Statistical Analysis**  Results were analyzed using IBM Statistical SPSS Program version 21.0 (IBM, NY, USA). Data are expressed as values of mean±standard deviation (SD). Data were analyzed using IBM SPSS Statistics 22 program and expressed as values of mean±standard deviation (SD). Statistical significance was analyzed using one−way analysis of variance (ANOVA) with post hoc Tukey−Kramer, for which *p <* .05 was considered significant. When data were not distributed normally, non−parametric tests were used to determine significance. |
| --- |

# REFERENCES

Amer, J., M. Dana and E. Fibach (2010). "The antioxidant effect of erythropoietin on thalassemic blood cells." Anemia **2010**: 978710.

Cao, A. and R. Galanello (2010). "Beta-thalassemia." Genet Med **12**(2): 61-76.

Cappellini, M. D. and I. Motta (2017). "New therapeutic targets in transfusion-dependent and -independent thalassemia." Hematology Am Soc Hematol Educ Program **2017**(1): 278-283.

Chacko, S. M., P. T. Thambi, R. Kuttan and I. Nishigaki (2010). "Beneficial effects of green tea: a literature review." Chin Med **5**: 13.

Clarke, K. A., T. P. Dew, R. E. Watson, M. D. Farrar, S. Bennett, A. Nicolaou, L. E. Rhodes and G. Williamson (2014). "High performance liquid chromatography tandem mass spectrometry dual extraction method for identification of green tea catechin metabolites excreted in human urine." J Chromatogr B Analyt Technol Biomed Life Sci **972**: 29-37.

Dai, W., D. Xie, M. Lu, P. Li, H. Lv, C. Yang, Q. Peng, Y. Zhu, L. Guo, Y. Zhang, J. Tan and Z. Lin (2017). "Characterization of white tea metabolome: Comparison against green and black tea by a nontargeted metabolomics approach." Food Res Int **96**: 40-45.

Galanello, R. and R. Origa (2010). "Beta-thalassemia." Orphanet J Rare Dis **5**: 11.

Gelderman, M. P., J. H. Baek, A. Yalamanoglu, M. Puglia, F. Vallelian, B. Burla, J. Vostal, D. J. Schaer and P. W. Buehler (2015). "Reversal of hemochromatosis by apotransferrin in non-transfused and transfused Hbbth3/+ (heterozygous B1/B2 globin gene deletion) mice." Haematologica **100**(5): 611-622.

Kautz, L., G. Jung, X. Du, V. Gabayan, J. Chapman, M. Nasoff, E. Nemeth and T. Ganz (2015). "Erythroferrone contributes to hepcidin suppression and iron overload in a mouse model of β-thalassemia." Blood **126**(17): 2031-2037.

Kautz, L. and E. Nemeth (2014). "Molecular liaisons between erythropoiesis and iron metabolism." Blood **124**(4): 479-482.

Khan, N. and H. Mukhtar (2013). "Tea and health: studies in humans." Curr Pharm Des **19**(34): 6141-6147.

Koonyosying, P., S. Kongkarnka, C. Uthaipibull, S. Svasti, S. Fucharoen and S. Srichairatanakool (2018). "Green tea extract modulates oxidative tissue injury in beta-thalassemic mice by chelation of redox iron and inhibition of lipid peroxidation." Biomed Pharmacother **108**: 1694-1702.

Makis, A., E. Voskaridou, I. Papassotiriou and E. Hatzimichael (2021). "Novel Therapeutic Advances in beta-Thalassemia." Biology (Basel) **10**(6).

Mandel, S. A., Y. Avramovich-Tirosh, L. Reznichenko, H. Zheng, O. Weinreb, T. Amit and M. B. Youdim (2005). "Multifunctional activities of green tea catechins in neuroprotection. Modulation of cell survival genes, iron-dependent oxidative stress and PKC signaling pathway." Neurosignals **14**(1-2): 46-60.

Matte, A., E. Federti, C. Kung, P. A. Kosinski, R. Narayanaswamy, R. Russo, G. Federico, F. Carlomagno, M. A. Desbats, L. Salviati, C. Leboeuf, M. T. Valenti, F. Turrini, A. Janin, S. Yu, E. Beneduce, S. Ronseaux, I. Iatcenko, L. Dang, T. Ganz, C. L. Jung, A. Iolascon, C. Brugnara and L. De Franceschi (2021). "The pyruvate kinase activator mitapivat reduces hemolysis and improves anemia in a beta-thalassemia mouse model." J Clin Invest **131**(10).

Pagani, A., A. Nai, L. Silvestri and C. Camaschella (2019). "Hepcidin and Anemia: A Tight Relationship." Front Physiol **10**: 1294.

Rivella, S. (2012). "The role of ineffective erythropoiesis in non-transfusion-dependent thalassemia." Blood Rev **26 Suppl 1**(0 1): S12-15.

Rivella, S. (2019). "Iron metabolism under conditions of ineffective erythropoiesis in beta-thalassemia." Blood **133**(1): 51-58.

Saewong, T., S. Ounjaijean, Y. Mundee, K. Pattanapanyasat, S. Fucharoen, J. B. Porter and S. Srichairatanakool (2010). "Effects of green tea on iron accumulation and oxidative stress in livers of iron-challenged thalassemic mice." Med Chem **6**(2): 57-64.

Settakorn, K., S. Hantrakool, T. Petiwithayakorn, N. Chalawturm, A. Tantiworawit, S. Fucharoen, S. Srichairatanakool and P. Koonyosying (2022). Consumption of green tea extract tablets improved anemia and decreased lipid-peroxidation product and nonheme iron contents of red cell membrane in transfusion-dependent beta-thalassemia patients. 3rd European Food Chemistry Congress. Montreal, Canada.

Settakorn, K., S. Kongkarnka, A. Chompupoung, S. Svasti, S. Fucharoen, J. B. Porter, S. Srichairatanakool and K. Koonyosying (2022). "Effects of green tea extract treatment on erythropoiesis and iron parameters in iron-overloaded β-thalassemic mice " Front Physiol **1-21**: 329-337.

Taher, A. T. and A. N. Saliba (2017). "Iron overload in thalassemia: different organs at different rates." Hematology Am Soc Hematol Educ Program **2017**(1): 265-271.

Taher, A. T., D. J. Weatherall and M. D. Cappellini (2018). "Thalassaemia." Lancet **391**(10116): 155-167.

Theodorou, A., M. Phylactides, L. Forti, M. R. Cramarossa, P. Spyrou, R. Gambari, S. L. Thein and M. Kleanthous (2016). "The investigation of resveratrol and analogs as potential inducers of fetal hemoglobin." Blood Cells Mol Dis **58**: 6-12.

Thephinlap, C., S. Ounjaijean, U. Khansuwan, S. Fucharoen, J. B. Porter and S. Srichairatanakool (2007). "Epigallocatechin-3-gallate and epicatechin-3-gallate from green tea decrease plasma non-transferrin bound iron and erythrocyte oxidative stress." Med Chem **3**(3): 289-296.

Upanan, S., K. Pangjit, C. Uthaipibull, S. Fucharoen, A. T. McKie and S. Srichairatanakool (2015). "Combined treatment of 3-hydroxypyridine-4-one derivatives and green tea extract to induce hepcidin expression in iron-overloaded β-thalassemic mice." Asian Pacific Journal of Tropical Biomedicine **5**(12): 1010-1017.

Viprakasit, V. and R. Origa (2014). Genetic basis, pathophysiology and diagnosis. Guidelines for the Management of Transfusion Dependent Thalassaemia (TDT). M. D. Cappellini, A. Cohen, J. Porter, A. Taher and V. Viprakasit. Nicosia (CY), Thalassaemia International Federation © 2014 Thalassaemia International Federation.
