# Supplementary material for: A randomized placebo−controlled clinical trial of oral green tea epigallocatechin 3−gallate on erythropoiesis and oxidative stress in transfusion−dependent β−thalassemia patients
Source: Front Mol Biosci. 2024 Jan 24;10:1248742. doi: 10.3389/fmolb.2023.1248742 (PMC10848917; doi:10.3389/fmolb.2023.1248742)
Supplement: Supplementary file 2 [file DataSheet1.docx]

Supplementary Data

A randomized placebo−controlled clinical trial of oral green tea epigallocatechin 3−gallate on erythropoiesis and oxidative stress in transfusion−dependent β−thalassemia patients

**Kornvipa Settakorn^1^, Sasinee Hantrakool^2,3^, Touchwin Petiwathayakorn^1^, Adisak Tantiworawit^2,3^, Pimlak Charoenkwan^4^, Nopphadol Chalortham^5^, Anchan Chompupoung^6^, Narisara Paradee^1^, Pimpisid Koonyosying^1^, Somdet Srichairatanakool^1*^**

*** Correspondence:**Somdet Srichairatanakool
[somdet.s@cmu.ac.th](mailto:somdet.s@cmu.ac.th)

**Supplementary Figure**


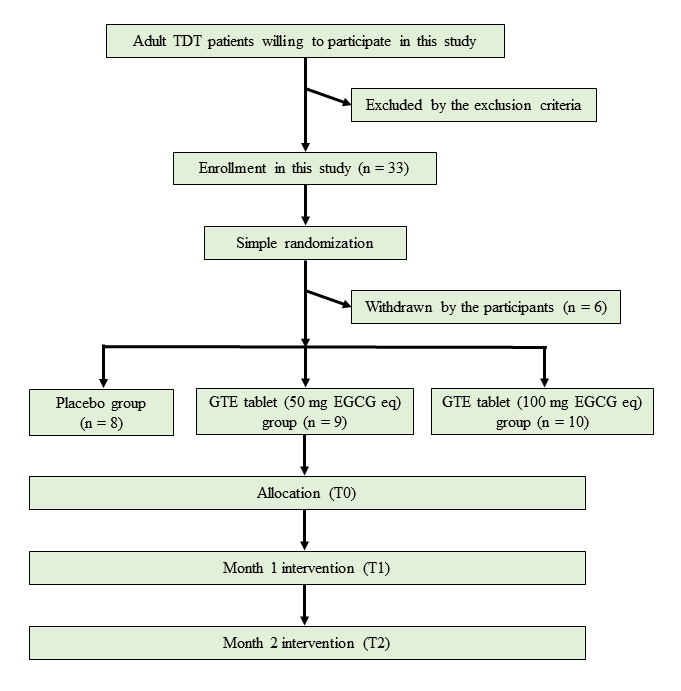


**Figure S1.** Diagram illustrating the participant selection process, the process employed for randomization and the intervention study design method used in this study for the selection of participants with thalassemia.
